# The Hsp90 Inhibitor, Monorden, Is a Promising Lead Compound for the Development of Novel Fungicides

**DOI:** 10.3389/fpls.2020.00371

**Published:** 2020-04-02

**Authors:** Hang T. T. Nguyen, Soyoung Choi, Soonok Kim, Ju-Hee Lee, Ae Ran Park, Nan Hee Yu, Hyeokjun Yoon, Chang-Hwan Bae, Joo Hong Yeo, Gyung Ja Choi, Hokyoung Son, Jin-Cheol Kim

**Affiliations:** ^1^Department of Agricultural Chemistry, Institute of Environmentally Friendly Agriculture, College of Agriculture and Life Science, Chonnam National University, Gwangju, South Korea; ^2^Department of Agricultural Biotechnology, Seoul National University, Seoul, South Korea; ^3^Biological and Genetic Resources Assessment Division, National Institute of Biological Resources, Incheon, South Korea; ^4^GPS Screen Team, Drug R&D Institute, Bioneer Corporation, Daejeon, South Korea; ^5^Therapeutic & Biotechnology Division, Center for Eco-friendly New Materials, Korea Research Institute of Chemical Technology, Daejeon, South Korea

**Keywords:** antifungal activity, disease control efficacy, Hsp90, mode of action, monorden

## Abstract

Endophytic fungi are great resources for the identification of useful natural products such as antimicrobial agents. In this study, we performed the antifungal screening of various plant endophytic fungi against the dollar spot pathogen *Sclerotinia homoeocarpa* and finally selected *Humicola* sp. JS-0112 as a potential biocontrol agent. The bioactive compound produced by the strain JS-0112 was identified as monorden known as an inhibitor of heat shock protein 90 (Hsp90). Monorden exhibited strong antagonistic activity against most tested plant pathogenic fungi particularly against tree pathogens and oomycetes with the minimum inhibitory concentration values less than 2.5 μg mL^–1^. Extensive *in planta* assays revealed that monorden effectively suppressed the development of several important plant diseases such as rice blast, rice sheath blight, wheat leaf rust, creeping bentgrass dollar spot, and cucumber damping-off. Especially, it showed much stronger disease control efficacy against cucumber damping-off than a synthetic fungicide chlorothalonil. Subsequent molecular genetic analysis of fission yeast and *Fusarium graminearum* suggested that Hsp90 is a major inhibitory target of monorden, and sequence variation among fungal Hsp90 is a determinant for the dissimilar monorden sensitivity of fungi. This is the first report dealing with the disease control efficacy and antifungal mechanism of monorden against fungal plant diseases and we believe that monorden can be used as a lead molecule for developing novel fungicides with new action mechanism for the control of plant diseases caused by fungi and oomycetes.

## Introduction

Plant pathogenic fungi are major causative agents of important plant diseases that threaten the agricultural industry worldwide (Dean et al., 2012). Until now, the control of fungal plant diseases largely depends on synthetic fungicides. However, the long term usage of the chemical fungicides has caused various negative effects such as residual toxicity, environment pollution, phytotoxicity, and the occurrences of drug-resistant pathogenic fungi to commercial fungicides (Brent and Hollomon, 1995; Parnell et al., 2006). Hence, the discovery of new natural metabolites and the development of novel fungicides using the natural metabolites as lead molecules are largely necessary in these days. Ideal lead molecules and fungicides would be highly effective against target pathogenic fungi, have new action mechanisms, and be safer to humans and the environment.

Among antagonistic microorganisms, use of endophytes has been considered as a potential option to substitute to agrochemicals on plant disease management (Ding et al., 2008; Kumar and Kaushik, 2012; Goudjal et al., 2014; De Silva et al., 2019). Endophytic bacteria and fungi are symbiotic microorganisms living inside healthy plant tissues without causing any disease symptoms. Some beneficial endophytes stimulate plant growth by producing plant hormones such as indole-3-acetic acid (Santoyo et al., 2016; Hassan, 2017), improve the tolerance to abiotic stresses (Pandey et al., 2018), or promote the plant immunity against microbial pathogens, insects, and herbivores (Azevedo et al., 2000; Ryan et al., 2008; Li et al., 2015; Bamisile et al., 2018).

Endophytic fungi exhibit the wide diversity that makes them to be great resources for the identification of useful natural products such as antimicrobial agents (Strobel and Daisy, 2003). Several bioactive secondary metabolites that suppress the plant diseases have been known to be produced by fungal endophytes. An oxygenated cyclohexanone derivative ([4S, 5S, 6S]-5,6-epoxy-4-hydroxy-3-ethoxy-5-methyl-cyclohex-2-en-1-one) isolated from an endophytic fungus *Amphirosellinia nigrospora* showed antifungal and antibacterial activities against various plant pathogens (Nguyen et al., 2019). Several compounds isolated from fungal endophytic *Phoma* species also exhibited antifungal activities (Wang et al., 2012). These studies demonstrated that endophytic fungi and their metabolites could be prospective resources for the development of novel plant disease control strategies.

The genus *Humicola*, previously known as an asexual genus in the *Chaetomiaceae*, was recently redefined to produce both asexual and sexual spores (Wang et al., 2019). Most of *Humicola* species were commonly found in soil, indoor environment, and composts whereas some of them have been known as endophytes (Radhakrishnan et al., 2015; Wang et al., 2016, 2019). *Humicola* species have displayed potential on the production of antibiotics that are appropriately used in human medicine and agriculture. Xanthoquinodins showing anticoccidial activity was isolated from soil *Humicola* sp. FO-888 (Tabata et al., 1993). *Humicola fuscoatra* KMM 4629 produced fuscoatrol A, 11-epiterpestacin, and β-nitropropionic acid, which showed antimicrobial activity against *Staphylococcus aureus* and *Bacillus subtilis* (Smetanina et al., 2004). *H. phialophoroides* exhibited potential suppression effect on fungal plant diseases (Ko et al., 2011). Fuscoatroside and fuscoatramide produced by *H. fuscoatra* NRRL 22980 showed antifungal activities against *Aspergillus flavus* (Joshi et al., 2002). This mycoparasite fungus *H. fuscoatra* NRRL 22980 also produced monorden, monocillin IV, and cerebrosides (Wicklow et al., 1998).

Monorden was first isolated from *Monosporium bonorden* as antifungal substance in the early 1950s (Delmotte and Delmotte-Plaquee, 1953). This compound displayed an inhibitory effect on growth and proliferation of fungal pathogens as well as cancer tumor (Sharma et al., 1998; Prodromou et al., 2009; Wicklow et al., 2009). Monorden showed significant biological control of antitumor in animal cells only in *in vitro*, but not in *in vivo* (Soga et al., 1999). Even though *in vitro* antifungal activity of this metabolite against fungal plant pathogens have been previously described well in the previous papers (Wicklow et al., 1998, 2009; Fujita et al., 1999), its disease control has not been reported till now. As for the target site of monorden, it was reported to bind to ATP-binding pocket in *N*-terminal domain of Hsp90 and then block its activity in cancer cells (Roe et al., 1999). Additionally, Prodromou et al. (2009) reported that replacement of isoleucine instead of leucine in binding site of Hsp90 reduced the affinity of monorden and this chaperone in a yeast model. However, its antifungal mechanism against fungal plant pathogens has not been studied.

Dollar spot is caused by the ascomycete fungus *Sclerotinia homoeocarpa* and is the most economically important turfgrass disease worldwide (Vargas, 1994). Management of this plant disease have largely been dependent on chemical fungicides such as benzimidazole, dicarboximide, and demethylase inhibitors. However, application of these chemicals is gradually being restricted because of the development of drug-resistant field strains and environmental concerns (Burpee, 1997). Alternative control strategies have been proposed to control dollar spot disease using suppressive composts, antagonistic fungi or bacteria, and antifungal natural products (Lo et al., 1997; Rodriguez and Pfender, 1997; Boulter et al., 2002).

In this study, we performed the massive antifungal screening of endophytic fungi against the dollar spot pathogen *S. homoeocarpa* and finally selected *Humicola* sp. JS-0112. Bioactive metabolite was revealed as monorden and this compound showed various antifungal activities against *S. homoeocarpa* as well as other plant pathogenic fungi and oomycetes. Therefore, this study aims (1) to isolate the fungal endophyte and its antifungal metabolite which could be used for the control of the dollar spot disease, (2) to evaluate the efficacy of the identified antifungal metabolite against other plant pathogenic fungi and oomycetes, and (3) to understand the molecular mechanism underlying the antifungal activity of the compound.

## Materials and Methods

### Microbial Species and Growth Conditions

Phytopathogenic fungi and oomycetes (Supplementary Table S1) which were used to test antifungal spectrum of the metabolite isolated from *Humicola* sp. JS-0112 were maintained on potato dextrose agar (PDA, Becton Dickinson, Sparks, MD, United States), except *Ophiostoma ulmi* which was grown on malt extract agar (MEA, Becton Dickinson) and *Phytophthora* species which were maintained on V8 agar (V8A) slants at 4°C. *Humicola* sp. JS-0112 was isolated from the root of *Ixeris repens* and maintained on PDA slants at 4°C. The fungal strain was inoculated in potato dextrose broth (PDB, Becton Dickinson) for 14 days at 25°C and 150 rpm for production of active compound. All fungal strains were cryogenic stored in glycerol 20% at −80°C. *F. graminearum* Z-3639 (Bowden and Leslie, 1999) was used as a parent strain for the construction of transgenic strains (Supplementary Table S2). *F. graminearum* transgenic strains, Δ*mat2* and HK226 (*FgHSP90:P_*ZEAR*_-FgHSP90*), were obtained from previous studies (Lee et al., 2003; Bui et al., 2016).

### Isolation and Identification of Endophytic Fungal Strains

Endophytic fungal strains were isolated, sequenced in the ribosomal region for putative identification, and stored for further study according to the procedures described in Nguyen et al. (2019). Each isolate was stored at −80°C as a glycerol stock before use, and deposited at the Wildlife Genetic Resources Bank of National Institute of Biological Resources (Supplementary Table S3). Preliminary identification of the endophytic fungi was based on cultural morphology on PDA and ITS sequences (Nguyen et al., 2019).

For the identification of the fungi showing strong antifungal activities, genomic DNA was extracted from the mycelial segments grown on PDA plates with Qiagen Plant DNA extraction kit (Qiagen, Hilden, Germany). Internal transcribed spacer (ITS) and large subunit of ribosomal DNA (LSU) was amplified with ITS1F (Gardes and Bruns, 1993) and LR5 (Vilgalys and Hester, 1990). Sequencing was conducted using five primers, ITS1F, ITS3, ITS4, LR0R, and LR5 (Vilgalys and Hester, 1990; White et al., 1990; Gardes and Bruns, 1993), to obtain sequences overlapped at least by two primers. Amplified fragments were purified and sequenced at Macrogen, Inc. (Seoul, South Korea). High quality sequences were aligned using Sequencer 5.0 (Ann Arbor, MI, United States) to obtain consensus sequences. BLAST searches were conducted against NCBI nr database and *ad hoc* BLAST DB. ITS and LSU sequences seen in the BLAST hits and related taxa were retrieved from NCBI. Multiple alignment was conducted with Clustal W, and subsequent phylogenetic analysis was carried out using MEGA version 7 (Tamura and Nei, 1993; Kumar et al., 2018). ITS and LSU sequences from the JS-0112 strain were deposited in NCBI GenBank under the accession number of MN006389 and MN006390, respectively.

### *In vitro* Screening of Antifungal Activities

The antagonistic activity of endophytic fungi isolated from the plant was assessed by 96 well microtiter plate using broth dilution method against *S. homoeocarpa*, a fungal pathogen of dollar spot of creeping bentgrass (Nguyen et al., 2019). The mycelial fragment suspensions in sterile distilled water (50 mg fresh mycelia per ml), at 1%, were used as inocula for antifungal bioassay using PDB.

The endophytic fungal strains were incubated in PDB for 14 days at 25°C and 150 rpm. The fermentation broth was filtered through 4-layer gauze and the filtrate was passed through a 0.2 μm membrane filter to get axenic culture filtrate for bioassay. The mycelia were extracted with acetone and then filtered through filter paper. The acetone filtrate was concentrated to dryness using a rotary vacuum evaporator (N-1110, EYELA Co., Tokyo, Japan) and redissolved using methanol (MeOH) at a concentration of 50 mg mL^–1^.

Mycelial extracts (500, 250, 125, 62.5, 31.25, and 15.6 mg mL^–1^) and axenic culture filtrates (20, 10, 5, 2.5, 1.25, 0.63%) were added into each well containing mycelial suspension of each test pathogenic fungus and then diluted in serial 2-fold dilutions. The plates were incubated at 25°C for 4 days. Minimum inhibitory concentration (MIC) values were defined as the lowest concentration of the mycelial extracts or culture filtrates causing complete inhibition of mycelial growth of the tested fungus. The bioassay was repeated two times in triplicate for each sample against *S. homoeocarpa* at all test concentrations.

### Determination of Antifungal Activity by Dual-Culture Assay

**FIGURE 1:**
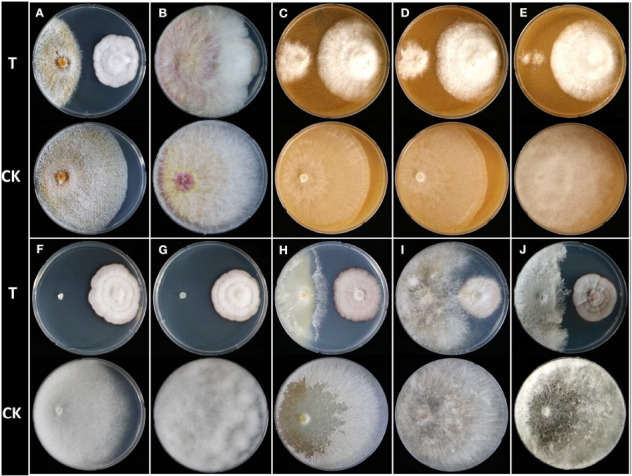
Dual culture assay of *Humicola* sp. JS-0112 against 10 phytopathogenic fungi and oomycetes. **(A)** *Cryphonectria parasitica*, **(B)** *Fusarium graminearum*, **(C)** *Phytophthora cambivora*, **(D)** *Phytophthora cactorum*, **(E)** *Phytophthora cinnamomi*, **(F)** *Pythium graminicola*, **(G)** *Pythium ultimum*, **(H)** *Raffaelea quercus-mongolicae*, **(I)** *Rhizoctonia solani*, **(J)** *Sclerotinia homoeocarpa*; T, co-cultivation of JS-0112 with phytopathogenic fungi; CK, pathogenic fungal strains cultivated alone served as controls.

To evaluate inhibitory activity of *Humicola* sp. JS-0112 against mycelial growth of 10 phytopathogenic fungi and oomycetes (Figure 1), dual culture assay was conducted as previously reported with minor modifications (Hamzah et al., 2018). Fungal disks of JS-0112 and pathogen (5 mm) were co-disposed at 5-cm distance on the PDA petri dish for most of the test fungi except *Phytophthora* species, which used V8 agar medium. The antifungal activity of JS-0112 was estimated by the inhibition of fungal growth of pathogen in comparison to a solely cultivated fungal agar disk. The fungal growth of pathogenic fungi was observed after 5–15 days after incubation at 25°C. The experiments were repeated twice in triplicate.

### Isolation of the Antifungal Metabolite

Six L of PDB cultures of JS-0112 were filtered through 4-layer gauze to separate culture filtrate and mycelia. The mycelia was extracted with acetone twice (1 L × 2), and then concentrated to dryness. The acetone extract was redissolved in 500 ml of distilled water and then partitioned with equal volumes of ethyl acetate (EtOAc) twice. The culture filtrate was also partitioned with EtOAc twice. The two EtOAc layers were combined and then concentrated to dryness using a rotary vacuum evaporator (N-1110, EYELA Co). The crude extract (14 g) was loaded onto silica gel (700 g, 70–230 mesh, 5 cm i.d × 60 cm; Merck, Darmstadt, Germany), which was eluted with chloroform:MeOH (99:1, v/v). Through thin layer chromatography analysis and *in vitro* bioassay using 96 well microtiter broth dilution method against *S. homoeocarpa*, the fractions showing antifungal activity were pooled and then further separated using a Sephedex LH-20 column (20 g, 2 cm i.d × 40 cm; Sigma-Aldrich Co., St. Louis, MO, United States) eluting progressively with 4:1 dichloromethane:hexane (120 mL) and 3:2 dichloromethane:acetone (220 mL), yielding the purified compound **1** (510 mg).

### Structural Elucidation of the Purified Compound

EI-MS and nuclear magnetic resonance spectroscopy (NMR) analyses were performed to determine the chemical structure of compound **1**. EI-MS was recorded on a JEOL JMS-700 high resolution mass spectrometer at 70 eV. ^1^H-NMR and ^13^C-NMR data of compound **1** were recorded using Bruker Avance III HD 500 MHz instrument (Bruker Biospin GmbH, Rheinstetten, Germany) in chloroform-d (Cambridge Isotope Laboratories, Inc., Andover, MA, United States) and tetramethylsilane (TMS) was used as an internal standard in the NMR analysis.

### *In vitro* Antifungal Activity of Monorden

The antifungal activity of monorden was evaluated by a serial broth dilution method as described above. Phytophathogenic fungi and oomycetes listed in Supporting Information Table S1 were used in this study. These pathogens were grown on PDB except *Ophiostoma ulmi* that was incubated on malt extract broth (MEB; MB Cell, South Korea). Monorden was dissolved in acetone at a concentration of 10 mg mL^–1^ as a stock solution, which was used to determine the MIC value against mycelia growth in 96 well-plate. Monorden was treated in a range of 0.078 – 100 μg mL^–1^. The final concentration of acetone was 1% v/v, and 1% acetone was used as an untreated control. All plates were incubated for 4–5 days at 25°C. MIC values were measured, and the experiment was repeated three times in triplicate against each fungal pathogen.

### Disease Control Efficacy of Monorden

To evaluate the disease control efficacy of monorden isolated from *Humicola* sp. JS-0112 against 9 plant diseases, the chemical was dissolved in acetone. The *in vivo* bioassays against 7 plant diseases including rice blast (RCB), rice sheath blight (RSB), tomato gray mold (TGM), tomato late blight (TLB), wheat leaf rust (WLR), barley powdery mildew (BPM), and red pepper anthracnose (RPA) were conducted as previously reported (Kim et al., 2001, 2004; Cho et al., 2006). Monorden was applied at 125, 250, and 500 μg mL^–1^ at 1 day before pathogen inoculation. Tween 20 solution (250 μg mL^–1^) containing 5% acetone was used as an untreated control and synthetic fungicides were used as positive controls (Table 1). The plant pots were randomly arranged with 3 replications per treatment and all experiments were repeated twice. Disease severity was measured 5 days after inoculation (DAI) for RCB, 8 DAI for RSB, 3 DAI for TGM, 4 DAI for TLB and RPA, 7 DAI for WLR and BPM. The mean values of the six estimates for each treatment were converted into control value.

**TABLE 1:** *In vitro* antifungal activity of monorden against mycelial growth of several phytopathogenic fungi.

**Phytopathogens**	**MIC^a^ value (μg mL^–1^)**
**Fungi**	
*Colletotrichum coccodes*	n.i.^b^
*Fusarium graminearum*	>100
*Fusarium oxysporum* f. sp. *lycopersici*	>100
*Botrytis cinerea*	50
*Magnaporthe oryzae*	50
*Rhizoctonia solani*	50
*Armillaria rolfsii*	50
*Botryosphaeria dothidea*	50
*Sclerotinia homoeocarpa*	25
*Raffaelea quercus-mongolicae*	25
*Ophiostoma ulmi*	12.5
*Cryphonectria parasitica*	1.56
*Taphrina wiesneri*	1.56
*Valsa kunzei*	1.56
**Oomycetes**	
*Phytophthora infestans*	2.5
*Phytophthora cactorum*	0.31
*Phytophthora capsici*	0.31
*Pythium ultimum*	0.31
*Phytophthora cinnamomi*	0.156
*Pythium helicolis*	0.156
*Pythium graminicola*	0.078
*Phytophthora cambivora*	0.078

*^*a*^MIC, minimum inhibitory concentration. ^*b*^n.i., no inhibition.*

The disease control efficacy of monorden was also tested against creeping bentgrass dollar spot (CDS). *S. homoeocarpa* was incubated in wheat-rice bran medium (wheat bran 200 mL, rice bran 100 mL, distilled water 100 mL in a 1-liter Erlenmeyer flask) for 7 days at 25°C. The culture was macerated for 10 s after adding 400 mL sterile distilled water containing 200 μg mL^–1^ streptomycin sulfate. The 3-week-old creeping bentgrass seedlings in vinyl pots (Φ 7 cm) were inoculated by adding 3.5 mL of the mycelial fragment suspension of *S. homoeocarpa* to soil. One hour after inoculation, these pots were treated with 10 mL of monorden solutions (250, 125, and 62.5 μg mL^–1^) by soil drench. A synthetic fungicide, Horikuo (tebuconazole 25%; Farmhannong Co., Ltd., Seoul, South Korea), at 2000-fold dilution was used as a positive control and Tween-20 (250 μg mL^–1^) solution containing 0.1% acetone was used as untreated control. The plants were grown at 25°C and relative humidity 95% for 7 days with 16 h light per day and then disease severity was assessed. Disease severity was determined as the percentage of infected aerial part. The experiment was carried out twice in triplicates and the mean values of the six estimates for each treatment were converted into control value.

The effect of monorden on the development of cucumber damping-off (CDO) caused by *Pythium ultimum* was examined by the previously established method with slight modifications (Li et al., 2007). In this study, the *in vivo* assay was done using 24-well microtiter plates (SPL Life Sciences Co., Ltd., Gyeonggi-do, South Korea). One cucumber seed was sown into each well containing approximately 2 g of sterile sand, and then agar plug of *P. ultimum* (Φ 5 mm) was inoculated onto seed, followed by covering with 1 g of sterile sand. Monorden was dissolved in acetone and then diluted with Tween 20 solution (250 μg mL^–1^) at concentrations of 100, 75, 50, 25, and 12.5 μg mL^–1^. An equal aliquot of 1 mL of each solution was treated into each well. A synthetic fungicide, chlorothalonil (Sigma-Aldrich Co) dissolved in acetone, was used as s positive control and treated at the same concentrations as monorden. Tween 20 solution containing 1% acetone was used as untreated control. The microtiter plates were incubated in a growth chamber for 7 days at 25°C with light of 16 h per day, and then post-emergence damping-off were observed. The experiment was conducted two times with three replications, each consisting of 10 seedlings. The mean values of the six estimates for each treatment were converted into control value.

### Drug Target Screening of Monorden in *Schizosaccharomyces pombe* Heterozygous Deletion Mutant Library

Drug target screening was performed using *S. pombe* heterozygous deletion mutants from Bioneer (South Korea). *S. pombe* heterozygous deletion mutant library was constructed as described before (Kim et al., 2010). Here, target screening of monorden was carried out using a Bioneer’s-unique drug-target screening system (*GPScreen*) at the GI_50_ of the agent on 94 genes of heat-shock protein-related functional group subset in *S. pombe* genome, using the principle of drug-induced haploinsufficiency (DIH) that measures growth inhibition by the compound on *S. pombe* heterozygous deletion mutants. The determination of GI_50_ of monorden in wild type *S. pombe* cells (SP286; h+/h+, ade6M210/ade6-M216, ura4-D18/ura4-D18, leu1-32/leu1-32) and the drug target screening were done with the same method as described (Lee et al., 2012). The cells were treated with the GI_50_ (4 μM) of monodern or with dimethylsulfoxide (DMSO)-only control after incubation at 30°C for 21 h. Fitness value which represents the growth-inhibitory potency of compound at GI_50_ dose in each deletion mutant was calculated by dividing cell mass (A_600_ nm) of DMSO control with the one of drug treated. Time-course assay was performed to confirm the cell growth inhibition in another way; after the compound treatment, cultures were incubated at 30 °C, and the cell mass (A_600_ nm) was recorded every 2 h using a microplate reader (M200Pro; Tecan, Maennedorf, Switzerland). Both assays were performed with three biological replicates.

### Construction of *F. graminearum* Transgenic Strains

To generate the *F. graminearum* transgenic strain expressing *HSP90* of *Cryphonectria parasitica*, the geneticin resistance gene cassette (*GEN*)*-P_*EF*__1α_* was amplified from the pSKGEN vector (Lee et al., 2011) using the pSKGEN-A and pSKGEN-C primers (Supplementary Table S4), and the *HSP90* open reading frame with 3′ region was amplified from genomic DNA of *C. parasitica* KACC40323 using Cp-A and Cp-C primers. The resulting two fragments were fused and the final construct was amplified using the nest primers pSKGEN-B and Cp-B. Final construct *GEN-P_*EF*1α_ -CpHSP90* was transformed into the *F. graminearum* wild type strain Z-3639 resulting in the *P_*EF*1α_ -CpHSP90* strain, of which *CpHSP90* is under the control of strong promoter *P_*EF*1α_* (Supplementary Table S2) as previously described (Son et al., 2011). Then heterothallic Δ*mat2* strain was outcrossed with *P_*EF*1α_ -CpHSP90* strain to generate the Δ*mat2*; *P_*EF*1α_ -CpHSP90* strain. Finally, the *FgHSP90:P_*ZEAR*_-FgHSP90*; *P_*EF*1α_ -CpHSP90* strain highly expressing *CpHSP90* but repressing *FgHSP90* was selected from an outcross Δ*mat2*; *P_*EF*1α_ -CpHSP90* × *P_*EF*1α_ -CpHSP90*. The PCR primers (Supplementary Table S4) used in this study were synthesized by an oligonucleotide synthesis facility (Bionics, Seoul, South Korea).

For outcross, mycelia of heterothallic female strains (Δ*mat2* or Δ*mat2*; *P_*EF*1α_ -CpHSP90*) grown on carrot agar plates for 5 days were fertilized with 1 mL of a conidial suspension from male strains. After sexual induction, all cultures were incubated under near-UV light (wavelength: 365 nm, Sankyo Denki Co., Ltd., Tokyo, Japan) at 25°C. Presence of *P_*ZEAR*_-FgHSP90* of the progeny from an outcross Δ*mat2*; *P_*EF*__1α_ -CpHSP90* × *P_*EF*1α_ -CpHSP90* was confirmed using a PCR assay with H3 and ZEAR-F3 primers (Supplementary Table S4).

### Statistical Analysis

Data on disease control efficacy of monorden against RCB, RSB, TGM, TLB, WLR, BPM, RPA, CDS, and CDO were analyzed separately using the statistical software SPSS ver. 23.0 (SPSS Inc., Chicago, IL, United States). All the experimental data are expressed as means ± standard deviation (SD) of replicates and investigated by one-way ANOVA using Duncan’s test. *p* < 0.05 was considered as statistically significant. The inhibition concentration (IC_50_) derived from monorden and chlorothalonil dose-response curves were determined using non-linear regression of GraphPad Prism 7 (Graph Pad Software Inc., San Diego, CA, United States).

## Results

### Antifungal Screening of Endophytic Fungi and Identification of JS-0112

We isolated 219 endophytic fungi from leaf, stem, or root tissues of 29 plant taxa in South Korea, and preliminary identification was carried out based on colony morphology on PDA and ITS sequence analysis (Supplementary Table S3). To screen potential biocontrol agents for the management of dollar spot caused by *S. homoeocarpa*, MIC values of both culture filtrates and mycelial extracts of 219 fungi against *S. homoeocarpa* were investigated. Among them, only JS-0112 and JS-0169 strains showed inhibitory effects against mycelial growth of *S. homoeocarpa* (Supplementary Table S3). Whereas the culture filtrate of JS-0169 displayed a weak *in vitro* antifungal activity with MIC value over 20%, both culture filtrate and mycelial extract of JS-0112 strongly inhibited the mycelial growth of *S. homoeocarpa* with MIC values of 1.25% and 125 μg mL^–1^, respectively. Antifungal activity of JS-0112 strain against *S. homoeocarpa* was also observed from the dual culture assay (Figure 1).

**FIGURE 2:**
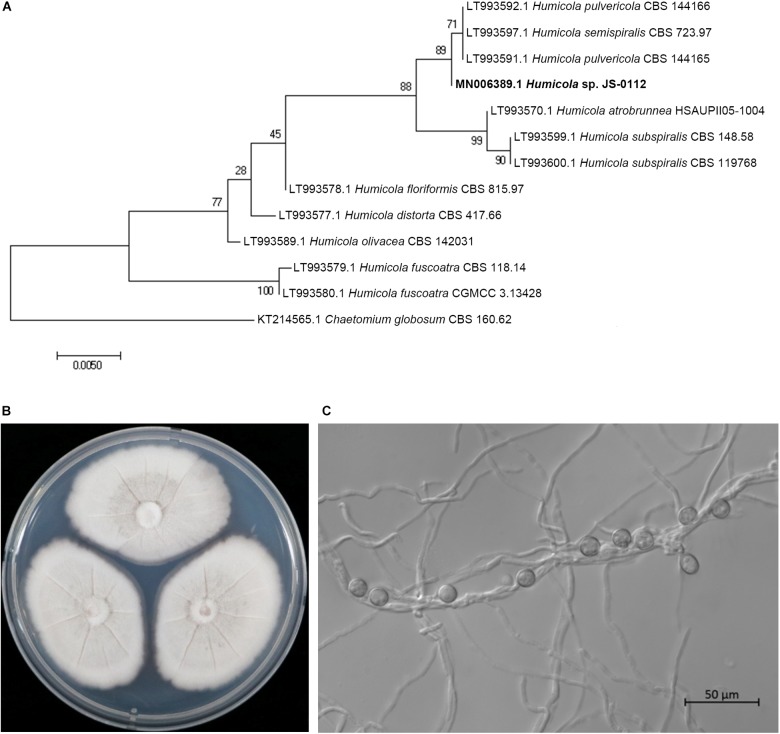
Morphological features and phylogenetic tree of JS-0112. **(A)** Phylogenetic tree reconstructed by Maximum Likelihood method with concatenated ITS and LSU sequences of JS-0112 and related taxa using MEGA version 7. GenBank accession number of each ITS-LSU sequences from each isolate was shown in front. ITS and LSU sequences of *Chaetomium globosum* strain CBS 160.62 were used as the outgroup. Bootstrap values with 1,000 replicates were illustrated on the node. **(B)** Colony morphology grown on PDA for 7 days. **(C)** Conidia produced on very short conidiophore along the hyphae. Bar indicates 50 μm.

The JS-0112 strain was identified as *Humicola* sp. that is closely related to *H. pulvericola* through phylogenetic analysis using concatenated ITS and LSU sequences (Figure 2A). Colonies on PDA after 7 days at 25°C were mouse gray to pale olivaceous; reverse pink to dull orange at the center and white at the margins (Figure 2B). JS-0112 produced pigmented, thick-walled and single-celled conidia on very short conidiophore along the hyphae, which is one of characteristics of genus *Humicola* (Wang et al., 2016, 2019; Figure 2C).

### Identification of Monorden and Its Control Efficacy for Creeping Bentgrass Dollar Spot (CDS)

The antifungal metabolite, compound 1, was purified from *Humicola* sp. JS-0112. Electron ionization mass spectrometry (EI-MS; 70 eV) of this compound appeared molecular ion peak at *m/z* 364, which inferred that the molecular formula is C_18_H_17_ClO_6_. ^1^H- and ^13^C-NMR data of monorden are presented in Supplementary Table S5. Compound 1 was identified as monorden by comparing with mass spectrometry (MS) and nuclear magnetic resonance spectroscopy (NMR) data reported by Wicklow et al. (1998), Yamamoto et al. (2003b), and Wicklow et al. (2009).

**FIGURE 3:**
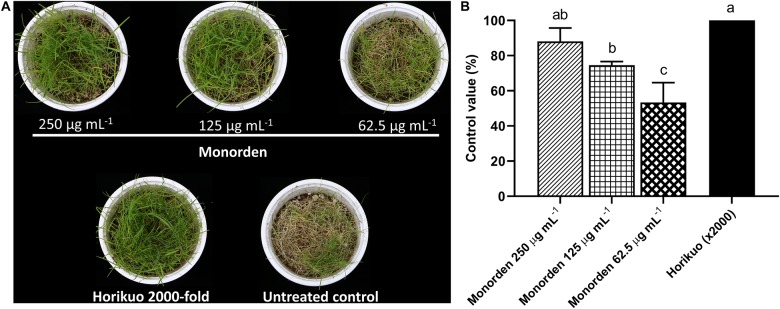
Disease control efficacy of monorden against dollar spot of creeping bentgrass caused by *Sclerotinia homoeocarpa*. **(A)** Symptoms of dollar spot on creeping bentgrass 7 days after inoculation. **(B)** Control values of monorden against creeping bentgrass dollar spot 7 days after inoculation. Disease severity was determined as the percentage of infected aerial part. Each value represents the means ± standard deviation of two runs with three replicates per run. Different lowercase letters show values that are significantly different (*p* < 0.05) level by Duncan’s test.

Then the disease control efficacy of monorden was tested against CDS. The results showed that the purified monorden alone was highly effective in reducing the development of CDS caused by *S. homoeocarpa*. Its control values were about 88, 75, and 53% at 250, 125, and 62.5 μg mL^–1^, respectively (Figures 3A,B). In particular, the CDS development was similarly suppressed by monorden 250 μg mL^–1^ compared to the synthetic chemical fungicide Horikuo (tebuconazole 25%).

### *In vitro* Antifungal Activity of JS-0112 and Monorden Against Various Fungal Pathogens

To explore the potential for use of JS-0112 or purified monorden in a broader range of fungal disease controls, we first performed the dual culture assay between JS-0112 and various plant pathogenic fungi and oomycetes (Supplementary Table S1 and Figure 1). Overall, *Humicola* sp. JS-0112 inhibited the mycelial growth of all tested plant pathogenic fungi and oomycetes (Figure 1). JS-0112 moderately retarded the radial growth of *Rhizoctonia solani*, and growth of *F. graminearum* was slightly delayed in dual culture assay with the JS-0112. Mycelial growth of *C. parasitica* and *R. quercus-mongolicae* as well as *S. homoeocarpa* was markedly inhibited when they were co-cultured with JS-0112. In particular, *Phytophthora* species were strongly sensitive to JS-0112, while mycelial growth of *Pythium* species was completely inhibited. The result suggests that antifungal activity of monorden is highly variable depending on fungal species.

We then investigated *in vitro* antifungal activity of monorden isolated from *Humicola* sp. JS-0112 against plant pathogenic fungi and oomycetes. Monorden inhibited the mycelial growth of all of the tested fungi and oomycetes with various MIC values except *Colletotrichum coccodes* (Table 1). Among the tested fungal species, tree pathogens (*C. parasitica*, *Taphrina wiesneri*, and *Valsa kunzei*) tend to be highly sensitive to monorden with MIC values of 1.56 μg mL^–1^, whereas *Fusarium* species (*F. graminearum* and *F. oxysporum* f. sp. *lycopersici*) showed moderate resistances toward monorden (Table 1). Intriguingly, most oomycetes were highly sensitive to monorden with MIC values of 0.078 – 2.5 μg mL^–1^; MIC values of most tested oomycetes were below than 0.39 μg mL^–1^ except *Phytophthora infestans* (2.5 μg mL^–1^). *Puccinia recondita* and *Blumeria graminis* which are causal agents for WLR and BPM, respectively, could not be used for *in vitro* experiment because they are obligate parasites.

### Disease Control Efficacy of Monorden Against Several Plant Diseases

We tested disease control efficacy of monorden isolated from *Humicola* sp. JS-0112 culture against seven plant diseases caused by fungi or oomycetes such RCB, RSB, TGM, TLB, WLR, BPM, and RPA (Table 2). Chemical fungicides widely used for each plant disease control were used for positive controls. Our virulence assays showed that monorden effectively suppressed the development of RCB caused by *Magnaporthe oryzae*, RSB caused by *R. solani* and WLR caused by *Puccinia recondita* in a dose-dependent manner. RCB was the most effectively controlled by monorden with control values of 90.0, 78.8, and 25.0% at 500, 250, and 125 μg mL^–1^, respectively. However, monorden was virtually inactive in reducing the development of TGM, TLB, BPM, and RPA (Table 2).

**TABLE 2:** Disease control efficacy of monorden against seven fungal plant diseases^a^.

**Chemical**	**Conc. (μg mL^–1^)**	**Control value (%)**						
		**RCB^b^**	**RSB**	**TGM**	**TLB**	**WLR**	**BPM**	**RPA**
Monorden	500	90.0ab^c^	75.0c	0.0d	0.0c	66.7b	0.0c	0.0c
	250	78.8b	45.0d	0.0d	0.0c	53.3c	0.0c	0.0c
	125	25.0c	40.0d	0.0d	0.0c	3.3d	0.0c	0.0c
Blasticidin-S	50	100.0a						
	1	78.8b						
Tricyclazole	10	100.0 a						
	0.5	97.5 a						
Validamycin	50		100.0a					
	5		90.0b					
Flutolanil	50		100.0a					
	20		100.0a					
Fludioxonil	50			100.0a				
	5			64.3c				
Fenhexamid	100			100.0a				
	20			78.6b				
Dimethomorph	10				100.0a			
	2				87.9b			
Chlorothalonil	100				100.0a			
	50				98.6a			
Flusilazole	10					100.0a		
	2					73.3b		
Pyraclostrobin	0.3					100.0a		
	0.1					93.3a		
Flusilazole	10						100.0a	
	0.5						86.7b	
Benomyl	100						100.0a	
	1						86.7b	
Dithianon	50							95.0a
	10							30.0b

*^*a*^The plant seedlings were inoculated with spores or mycelial suspensions of the pathogens 1 day after the chemical solutions were sprayed to run-off on the leaves. ^*b*^RCB, Rice blast by *M. oryzae*; RSB, rice sheath blight by *R. solani*; TGM, tomato gray mold by *B. cinerea*; TLB, tomato late blight by *P. infestans*; WLR, wheat leaf rust by *P. recondita*; BPM, barley powdery mildew by *B. graminis*; RPA, red pepper anthracnose by *C. coccodes*. ^*c*^Disease rating was conducted 3–8 days after inoculation. Means with same letter within a column are not significantly different (*p* < 0.05) by Duncan’s test.*

**FIGURE 4:**
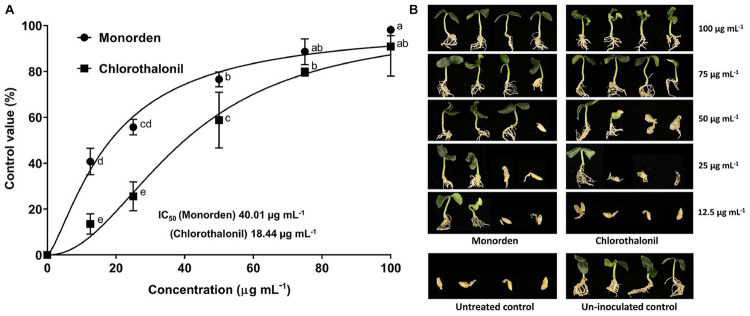
Disease control efficacy of monorden against cucumber damping-off (CDO) caused by *Pythium ultimum*. **(A)** Dose-response curve and IC50 values in the control of CDO. Each value represents the means ± standard deviation of two runs with three replicates per run. Each replicate consisted of ten seedlings. Data from two runs were plotted using Graph Pad Prism Version 7 (Graph Pad Software Inc., San Diego, CA, United States). Different small letters indicate significant different values (Duncan’s test, *p* < 0.05). **(B)** Post emergence damping-off of cucumber 7 days after inoculation.

Monorden very effectively reduced the development of CDO caused by *P. ultimum*. Monorden even showed higher control efficacy against CDO than a commercial fungicide, chlorothalonil (Figure 4). The calculated IC_50_ (the concentration that controls 50% of the disease severity) values were 18.44 μg mL^–1^ for monorden and 40.01 μg mL^–1^ for chlorothalonil.

### Drug-Induced Haploinsufficiency (DIH) Assay in the Fission Yeast *S. pombe*

Monorden has been known as an inhibitor of Hsp90, which is a chaperone protein embarking in protein folding and degradation (Sharma et al., 1998). In order to fully apprehend the mode of action of monorden in fungi, we exploited drug-induced haploinsufficiency (DIH) assay with the fission yeast *S. pombe* heterozygous deletion mutant library (Kim et al., 2010) and a high-throughput genome-wide drug target identification system (Lee et al., 2012). For this study, deletion mutants of 94 genes related to posttranslational modification, protein turnover, chaperones among *S. pombe* genome-wide deletion mutant library (4,845 genes) were used to screen the drug target of monorden (Supplementary Table S6).

**FIGURE 5:**
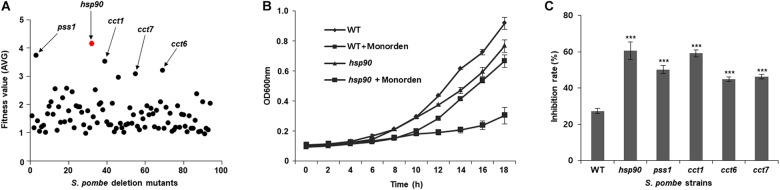
Identification of potential targets of monorden using the drug target screening system of a *S. pombe* mutant library. **(A)** Monorden was treated to culture of *S. pombe* genome-wide deletion library at the GI_50_ (4 μM) for 21 h at 30 °C and the fitness values of each mutant were plotted as the mean of three biological replicates (Supplementary Table S6). **(B)** Time-course assay was performed to confirm the cell growth inhibition of *S. pombe* mutants; after the compound treatment, cultures were incubated at 30 °C, and the cell mass (A_600_ nm) was recorded every 2 h. Data was presented as the mean and standard error of three biological replicates. **(C)** Inhibition rate of each *S. pombe* strains at 18 h after the treatment in **(B)** and Supplementary Figure S2 was calculated. ***p < 0.001.

Monorden showed potent anti-proliferative activity against wild-type *S. pombe* cells for 21 h (GI_50_ = 4 μM; Supplementary Figure S1). DIH by monorden in various *S. pombe* heterozygous deletion mutants revealed that fitness scores of five gene deletion mutants (*hsp90*, *pss1*, *cct1*, *cct6*, and *cct7*) were over 3.0 among the mutants that were inhibited by monorden at GI_50_ (Figure 5). For target validation, the wild-type strain and five deletion mutants were cultured and treated with either DMSO-only or monorden (4 μM). As the results, the cell growth of *hsp90*, *pss1*, and *cct1* mutants was markedly inhibited by monorden compared to wild type (WT) of *S. pombe* cells (Figures 5B,C, and Supplementary Figure S2). The results may demonstrate that Pss1, Cct1, Cct6, and Cct7 could be putative targets of monorden. However, *in silico* protein-protein network analysis using STRING (Szklarczyk et al., 2018) showed that putative targets of monorden identified in this study (Pss1, Cct1, Cct6, and Cct7) interact with Hsp90, demonstrating that they are not directly targeted by monorden (Supplementary Figure S3).

### Relationship Between Hsp90 Sequence Variation and Monorden Sensitivity

The result of the DIH assay demonstrates that fungal Hsp90 orthologs are major target for monorden, although some other chaperones are possibly inhibited by this compound. Supposing that structural differences among Hsp90 orthologs contribute to the dissimilar sensitivity against monorden in fungi and oomycetes, we first compared Hsp90 ortholog sequences. Phylogenetic and alignment analyses showed that Hsp90 orthologs of oomycetes are more variable and have distinct C-terminal regions compared to those of fungi (Supplementary Figures S4, S5). In particular, many *Fusarium* specific sequence regions were observed among fungal Hsp90 proteins, suggesting that structural alteration caused by these sequence variations is important for the resistant nature of *Fusarium* species against monorden (Supplementary Figure S6). All of the analyzed Hsp90 orthologs except that of JS-0112 strain do not have L34I mutation (Red asterisk in Supplementary Figure S6) which is responsible for the monorden resistance in monorden-producing fungus *H. fuscoatra* (Prodromou et al., 2009).

**FIGURE 6:**
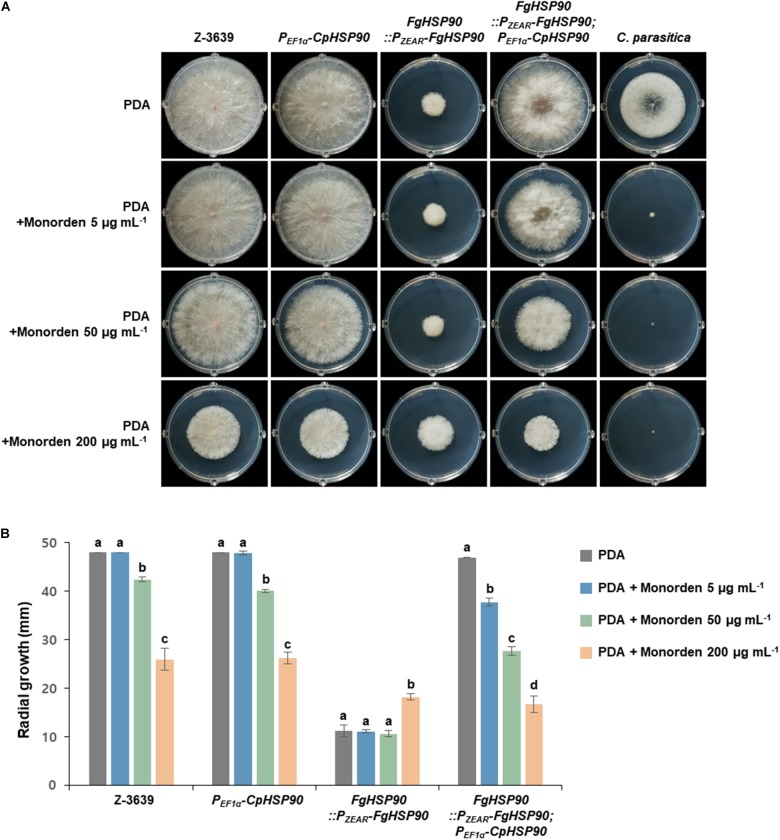
Mycelial growth of *F. graminearum* strains on PDA and PDA supplemented with monorden. **(A)** Colony morphology and **(B)** Radial growth of fungal strains. *F. graminearum* Z-3639, *Cryphonectria parasitica* KACC40323, and transgenic *F. graminearum* strains carrying *P_*EF1*α_ -CpHSP90* and/or *P_*ZEAR*_-FgHSP90* were inoculated on PDA and PDA supplemented with different concentrations of monorden (5, 50, and 200 μg mL^–1^). Data were obtained 4 days after inoculation. Three replicate plates of each medium per isolate were used and radial growth was measured three times for each plate. For each strain, same letters mean there is no significant difference among culture conditions, different letters represent statistically significant differences at *p* < 0.05.

To gather insights into the relationship between global Hsp90 sequence variation and monorden sensitivity, we applied the molecular genetics approach with two Hsp90 orthologs of *F. graminearum* and *C. parasitica* which showed the lowest and highest sensitivities toward monorden among tested fungi (Table 1). We hypothesized that the *F. graminearum* transgenic strain expressing *HSP90* of *C. parasitica* (*CpHSP90*) without its native *HSP90* (*FgHSP90*) would be more sensitive toward monorden than the wild-type strain. Since *HSP90* is essential gene in *F. graminearum*, we used *HSP90* knock-down mutant HK226 (*FgHSP90:P_*ZEAR*_-FgHSP90*) for this study (Bui et al., 2016). *P*_*ZEAR*_ is a zearalenone inducible promoter and therefore expression of *FgHSP90* remains very low without supplementation of zearalenone (Lee et al., 2010). The *FgHSP90:P_*ZEAR*_-FgHSP90*; *P_*EF*__1α_ -CpHSP90* strain similarly grew on PDA compared to the wild-type strain, suggesting that *CpHSP90* is the functional ortholog of *FgHSP90* (Figure 6).

We compared mycelial growth of *F. graminearum* strains on PDA with or without supplementation of monorden (Figure 6). The *FgHSP90* knock-down mutant (*FgHSP90:P_*ZEAR*_-FgHSP90*) showed reduced sensitivity to excess monorden (200 μg mL^–1^), whereas the *F. graminearum* wild-type strain were reduced in growth in a dose-dependent manner and showed about 50% growth reduction on PDA with 200 μg mL^–1^ monorden treatment. This result indicates that FgHsp90 is the primary inhibitory target of monorden although *F. graminearum* is highly resistant to monorden compared to other fungal species. When *CpHSP90* was expressed in *FgHSP90* knock-down mutant, the transgenic strain (*FgHSP90:P_*ZEAR*_-FgHSP90*; *P_*EF*__1α_ -CpHSP90*) was significantly reduced in growth even under 5 μg mL^–1^ monorden as *C. parasitica* (Figure 6). This is the clear evidence demonstrating that CpHsp90 is much more susceptible to monorden than FgHsp90 in the *F. graminearum* cell system.

## Discussion

The polyketide monorden was first isolated and identified in 1953 from a culture of *M. bonorden* (Delmotte and Delmotte-Plaquee, 1953) and named in 1964 (McCapra et al., 1964). Mirrington et al. (1964) also isolated this compound from the culture filtrate of *Nectria radicicola* and gave the name “Radicicol.” Monorden has been known to be produced by *Pochonia* species (Stadler et al., 2003), *Penicillium luteo-aurantium* (Nozawa and Nakajima, 1979), *Pochonia chlamydosporia* var. *catenulata* (Hellwig et al., 2003), *Chaetomium chiversii* (Turbyville et al., 2006), *Colletotrichum graminicola* (Wicklow et al., 2009), and several *Humicola* species (Wicklow et al., 1998; Fujita et al., 1999). Moreover, biological activities of monorden have already been described in previous reports (Wicklow et al., 1998; Fujita et al., 1999; Winssinger and Barluenga, 2007). This chemical is known to act as a selective Hsp90 inhibitor, and exhibits antifungal and antitumor properties (Ayer et al., 1980; Sharma et al., 1998; Wicklow et al., 2009). In particular, monorden has been shown to exert antimicrobial activity against the oomycete *Pythium debaryanum* with an MIC value of 10 μg mL^–1^ (lowest concentration evaluated) and the fungal pathogen *R. solani* (MIC = 100 μg mL^–1^) (Ayer et al., 1980). However, to the best of our knowledge, *in planta* assays of monorden activity in the context of fungal plant diseases have not been performed.

In this study, we found that endophytic fungus *Humicola* sp. JS-0112 produced monorden. In addition, extensive *in vitro* and *in planta* assays showed that monorden significantly suppressed mycelial growth and/or virulence of several economically important plant pathogenic fungi. Most fungal pathogens were highly sensitive to monorden while *Fusarium* species (*F. graminearum* and *F. oxysporum* f. sp. *lycopersici*) and *C. coccodes* showed moderate resistance to this compound (Table 1). In agreement with the results of our *in vitro* assays, monorden successfully reduced the development of RCB (*M. oryzae*), RSB (*R. solani*), WLR (*P. recondita*), and CDS (*S. homoeocarpa*) in a dose-dependent manner (Table 1). Although *B. cinerea* showed similar levels of sensitivity toward monorden when compared with *M. oryzae* and *R. solani* (MIC = 50 μg mL^–1^), and the mycelial growth of *P. infestans* was highly sensitive (MIC = 2.5 μg mL^–1^), monorden failed to reduce TGM (*B. cinerea*) and TLB (*P. infestans*) severity in infected plants. This means that the disease control efficacy of monorden depends not only on drug sensitivity but also on other factors including variable host physiologies or pathosystems. Interestingly, tree pathogenic fungi (*Botryosphaeria dothidea*, *C. parasitica*, *O. ulmi*, *R. quercus-mongolicae*, *T. wiesneri*, and *V. kunzei*) tend to be more sensitive to monorden than pathogens of crop or vegetable plants. Because the control of tree diseases is very tricky and largely dependent on chemical fungicides, further *in planta* studies of these tree pathogenic fungi could increase the likelihood of developing an industrial application for monorden in the forestry industry.

Together with plant pathogenic fungi, some oomycete-derived plant diseases have long been a source of concern in the agricultural industry (Kamoun et al., 2015). Although oomycetes are superficially similar to filamentous fungi, they are more closely related to algae and plants than fungi (Van de Peer and De Wachter, 1997). Therefore, fungi and oomycetes generally have quite different physiology including variations in sensitivity to synthetic fungicides and natural metabolites (Latijnhouwers et al., 2003). As the management of oomycete diseases primarily depends on chemical fungicides, the development of novel natural products with strong antagonism toward oomycetes remains an industry priority.

Several natural products demonstrating strong antagonistic effects on oomycetes have been reported. Chaetomin (MIC = 2.5 μg mL^–1^) and 4-phenyl-3-butenoic acid (MIC = 0.5 μg mL^–1^) have potent antimicrobial activity against *P. ultimum* (Di Petro et al., 1992; Lee et al., 2005). Xanthyletin (MIC = 5 μg mL^–1^) and 4-formylsyringol (MIC = 10 μg mL^–1^) were revealed to possess strong antagonistic effects against *Pythium insidiosum* (Suthiwong et al., 2017). Our comprehensive *in vitro* and *in planta* assays reveal that monorden is also a strong antagonistic agent of various oomycetes. Mycelial growth of oomycete species, except for *P. infestans* (MIC = 2.5 μg mL^–1^), was severely inhibited following treatment with monorden with MIC values of less than 0.31 μg mL^–1^ (Table 1). Moreover, the disease control efficacy of monorden against CDO—caused by *P. ultimum*—was much higher than that of the widely used chemical fungicide chlorothalonil (Figure 4). These data indicate that, to date, monorden and its derivatives could potentially be the most effective fungi-derived antimicrobial agents for the control of plant diseases caused by oomycetes.

Fungi and oomycetes show variable degrees of sensitivity toward monorden depending on the species. *Fusarium* species, in particular, possess efficient molecular mechanisms allowing for moderate monorden resistance while other tree pathogenic fungi and oomycetes were rendered inviable even at very low concentrations of monorden. The results of the DIH assays and heterologous expression experiments suggested that Hsp90 orthologs are the major inhibitory target for monorden in fungi even in the monorden-resistant fungus *F. graminearum*. Sequence discrepancies in the Hsp90 orthologs from different fungi seem to be the major determinants of the degree of susceptibility to monorden. Although Hsp90 orthologs of *Fusarium* species did not carry the L34I mutation (Prodromou et al., 2009), there are dozens of *Fusarium*-specific sequences. Moreover, oomycetes have distinct and dissimilar N- and C-terminal sequences compared to other fungal species (Supplementary Figures S5, S6). Since both N-terminal (IRP036890, HSP90-like ATPase) and C-terminal (IRP037196, HSP90, C-terminal domain) regions are important for the proper function of Hsp90 proteins, we suspect that structural changes derived from sequence variation might be important in determining the resistance profile of fungi and oomycetes toward monorden (Allan et al., 2006). Further in-depth molecular genetic analysis will focus on the characterization of the links between sequence variations in Hsp90 and monorden resistance in fungi and oomycetes.

Several metabolites have been synthesized from monorden in order to improve its stability and antitumor activity. Cycloproparadicicol, synthesized from monorden and cyclopropane, has been shown to have excellent antitumor activity when compared to monorden (Yamamoto et al., 2003a). Novel halohydrin and oxime derivatives of monorden were synthesized and some of these have been shown to have improved antitumor activities compared with their parental compound (Agatsuma et al., 2002). Likewise, we demonstrated that monorden could be used as a lead compound to develop effective fungicides for the treatment of plant pathogenic fungi and oomycetes. A number of fungicides for the control of fungal plant diseases have been developed and they can be divided into four groups based on their mode of action: sterol synthesis inhibitors, mitochondrial electron transport inhibitors, multi-site enzyme inhibitors, nucleic acid and protein synthesis inhibitors (Tillman et al., 1973; Berg et al., 1986; Bartlett et al., 2002). To the best of our knowledge, there is no commercial fungicide with Hsp90 inhibition as the mechanism of action.

## Conclusion

Conclusively, we proved that monorden itself is a strong antimicrobial agent for the effective control of several important plant diseases caused by fungi and oomycetes. Because structural changes of Hsp90 protein mainly affect monorden susceptibility, the approach of target-based drug discovery using monorden as a lead compound will be possible to develop novel fungicides for the control of plant diseases caused by fungi and oomycetes.

## Data Availability Statement

The datasets generated for this study can be found in the MN006389 and MN006390.

## Author Contributions

J-CK, HS, and HN designed and conceived the experiments. SC, HY, C-HB, and JY isolated the endophytic fungi and identified the strains. HN isolated and identified monorden. HN, AP, NY, and GC performed *in vitro* and *in vivo* antifungal bioassays. J-HL conducted the drug-induced haploinsufficiency (DIH) screening. SC and HS examined the relationship between Hsp90 sequence variation of different fungi and monorden sensitivity by using *Fusarium graminearum* transgenic strain expressing *HSP90* of *C. parasitica*. HN, SC, SK, J-HL, AP, NY, HY, C-HB, and JY analyzed the data. All authors contributed to interpretation of the results, read, and approved the final manuscript. HN, SC, HS, and J-CK wrote the manuscript with inputs from all authors.

## Conflict of Interest

The authors declare that the research was conducted in the absence of any commercial or financial relationships that could be construed as a potential conflict of interest.
