# Corrigendum: The Hsp90 Inhibitor, Monorden, Is a Promising Lead Compound for the Development of Novel Fungicides

**DOI:** 10.3389/fpls.2020.00635

**Published:** 2020-05-20

**Authors:** Hang T. T. Nguyen, Soyoung Choi, Soonok Kim, Ju-Hee Lee, Ae Ran Park, Nan Hee Yu, Hyeokjun Yoon, Chang-Hwan Bae, Joo Hong Yeo, Gyung Ja Choi, Hokyoung Son, Jin-Cheol Kim

**Affiliations:** ^1^Department of Agricultural Chemistry, Institute of Environmentally Friendly Agriculture, College of Agriculture and Life Science, Chonnam National University, Gwangju, South Korea; ^2^Department of Agricultural Biotechnology, Seoul National University, Seoul, South Korea; ^3^Biological and Genetic Resources Assessment Division, National Institute of Biological Resources, Incheon, South Korea; ^4^GPS Screen Team, Drug R&D Institute, Bioneer Corporation, Daejeon, South Korea; ^5^Therapeutic & Biotechnology Division, Center for Eco-friendly New Materials, Korea Research Institute of Chemical Technology, Daejeon, South Korea

**Keywords:** antifungal activity, disease control efficacy, Hsp90, mode of action, monorden

In the original article, there were mistakes in Figure 1C and Figure 4B as published. Figure 1D (*Phytophthora cactorum*) was duplicated in Figure 1C (*Phytophthora cambivora*) and two representative images for cucumber damping-off symptom were overlapped in Figure 4B. The corrected figures appear below.

**Figure 1:**
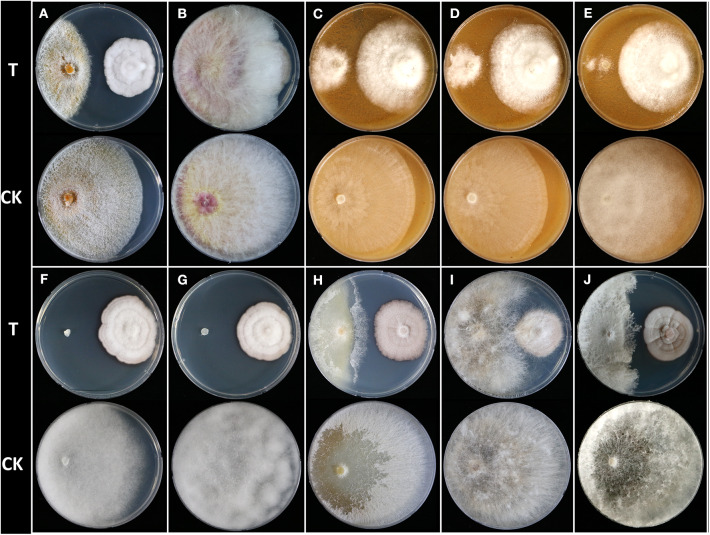
Dual culture assay of *Humicola* sp. JS-0112 against 10 phytopathogenic fungi and oomycetes. **(A)** *Cryphonectria parasitica*, **(B)** *Fusarium graminearum*, **(C)** *Phytophthora cambivora*, **(D)** *Phytophthora cactorum*, **(E)** *Phytophthora cinnamomi*, **(F)** *Pythium graminicola*, **(G)** *Pythium ultimum*, **(H)** *Raffaelea quercus-mongolicae*, **(I)** *Rhizoctonia solani*, **(J)** *Sclerotinia homoeocarpa*; T, co-cultivation of JS-0112 with phytopathogenic fungi; CK, pathogenic fungal strains cultivated alone served as controls.

**Figure 4:**
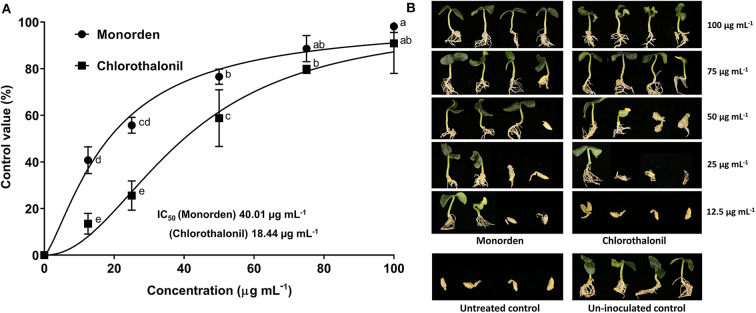
Disease control efficacy of monorden against cucumber damping-off (CDO) caused by *Pythium ultimum*. **(A)** Dose-response curve and IC50 values in the control of CDO. Each value represents the means ± standard deviation of two runs with three replicates per run. Each replicate consisted of ten seedlings. Data from two runs were plotted using Graph Pad Prism Version 7 (Graph Pad Software Inc., San Diego, CA, United States). Different small letters indicate significant different values (Duncan's test, *p* < 0.05). **(B)** Post emergence damping-off of cucumber 7 days after inoculation.

The authors apologize for this error and state that this does not change the scientific conclusions of the article in any way. The original article has been updated.

