# Supplementary material for: The Hsp90 Inhibitor, Monorden, Is a Promising Lead Compound for the Development of Novel Fungicides
Source: Front Plant Sci. 2020 Apr 2;11:371. doi: 10.3389/fpls.2020.00371 (PMC7144829; doi:10.3389/fpls.2020.00371)
Supplement: Supplementary file 1 [file Data_Sheet_1.docx]

Supplementary Material

# Supplementary Figures and Tables

## Supplementary Figures

**
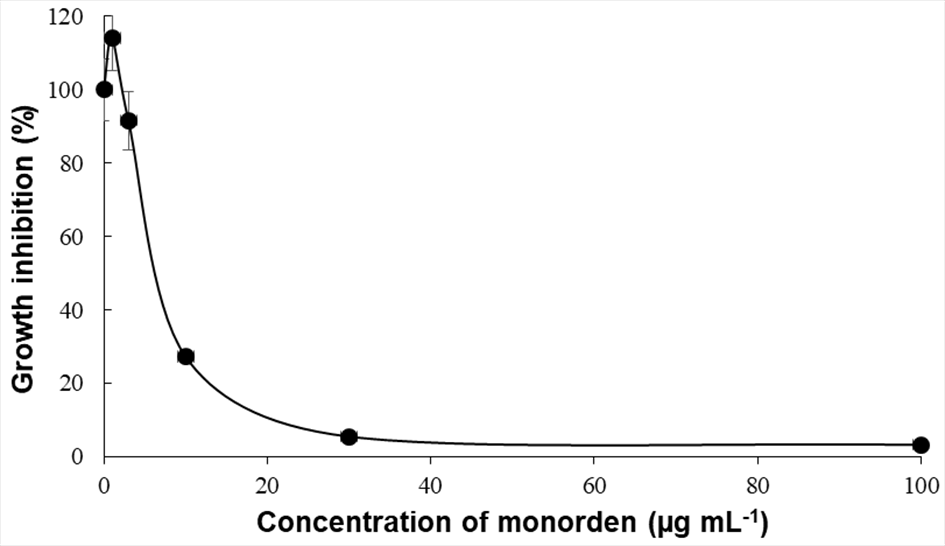
**

**Supplementary Figure S1 |** Growth-inhibitory activity (GI_50_) of monorden in wild type *S. pombe* cells (SP286).


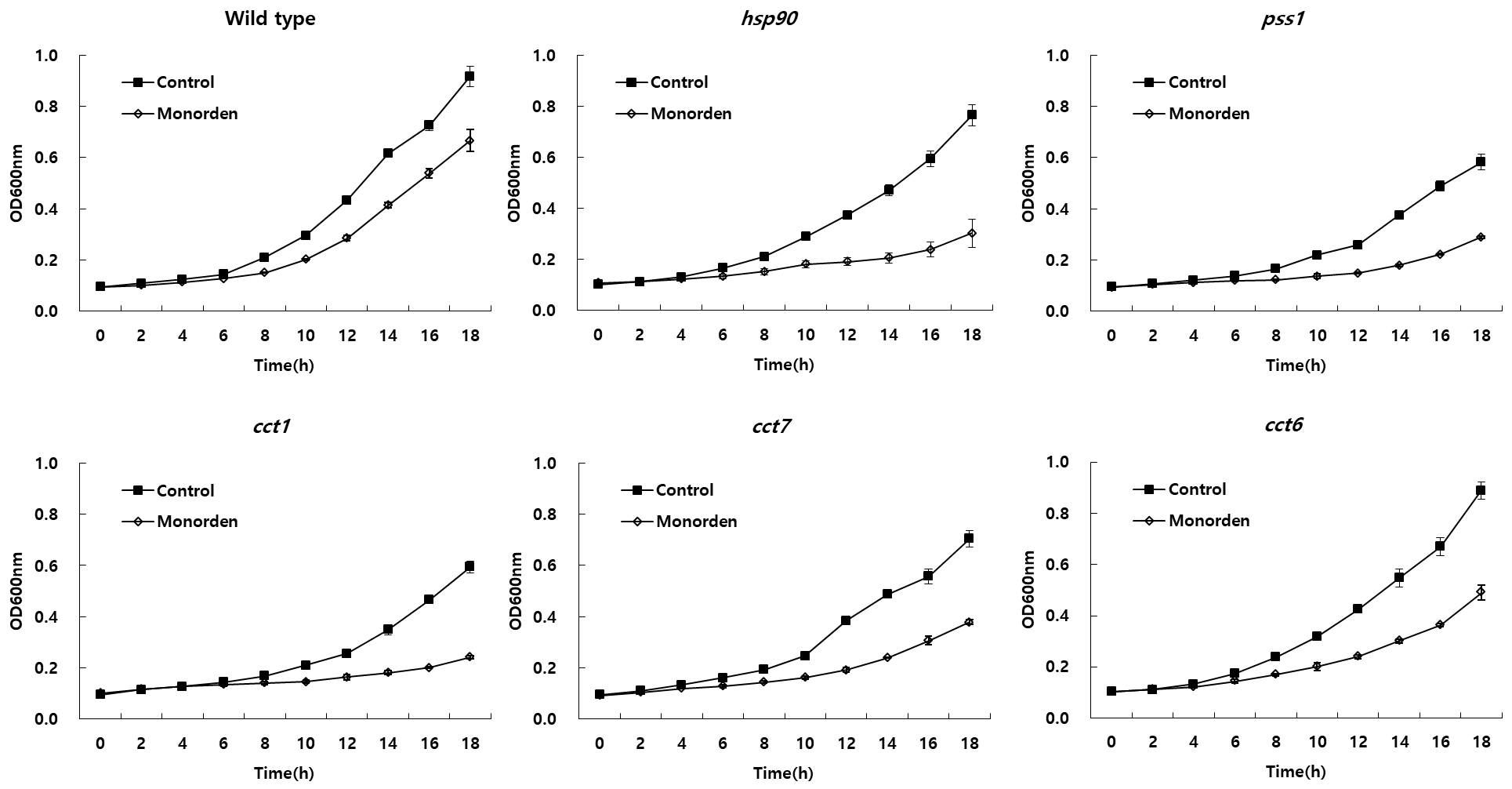


**Supplementary Figure S2 |** Time-course assay of *S. pombe* mutants. For target validation, the wild-type strain and the 5 target candidates showing fitness score > 3.0 were cultured and treated with either DMSO-only or monorden (4 μM). The cell mass was recorded every 2 h with spectrophotometry (OD_600nm_). Data was presented as the mean and standard error of three biological replicates.


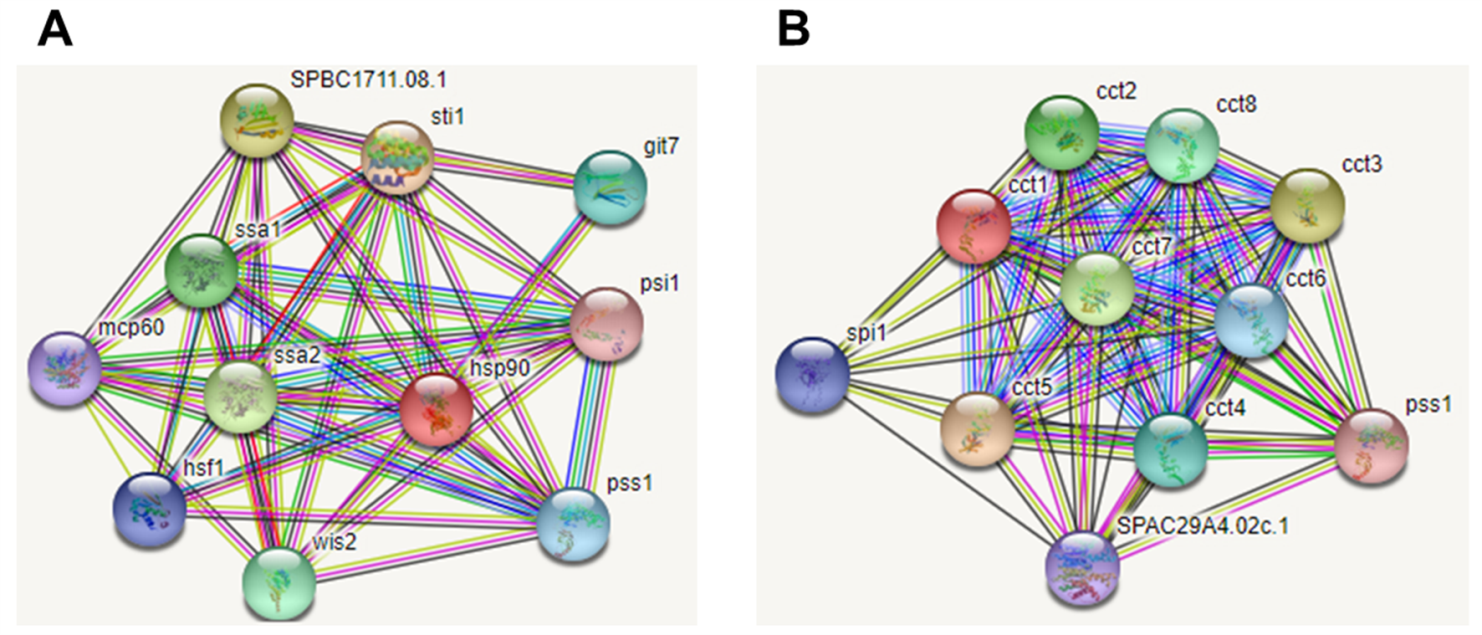


**Supplementary Figure S3 |** Protein-protein network of the linkage between the Hsp90 (A) or Cct1 (B) and their associated partners of *S. pombe* in STRING database (http://string-db.org).


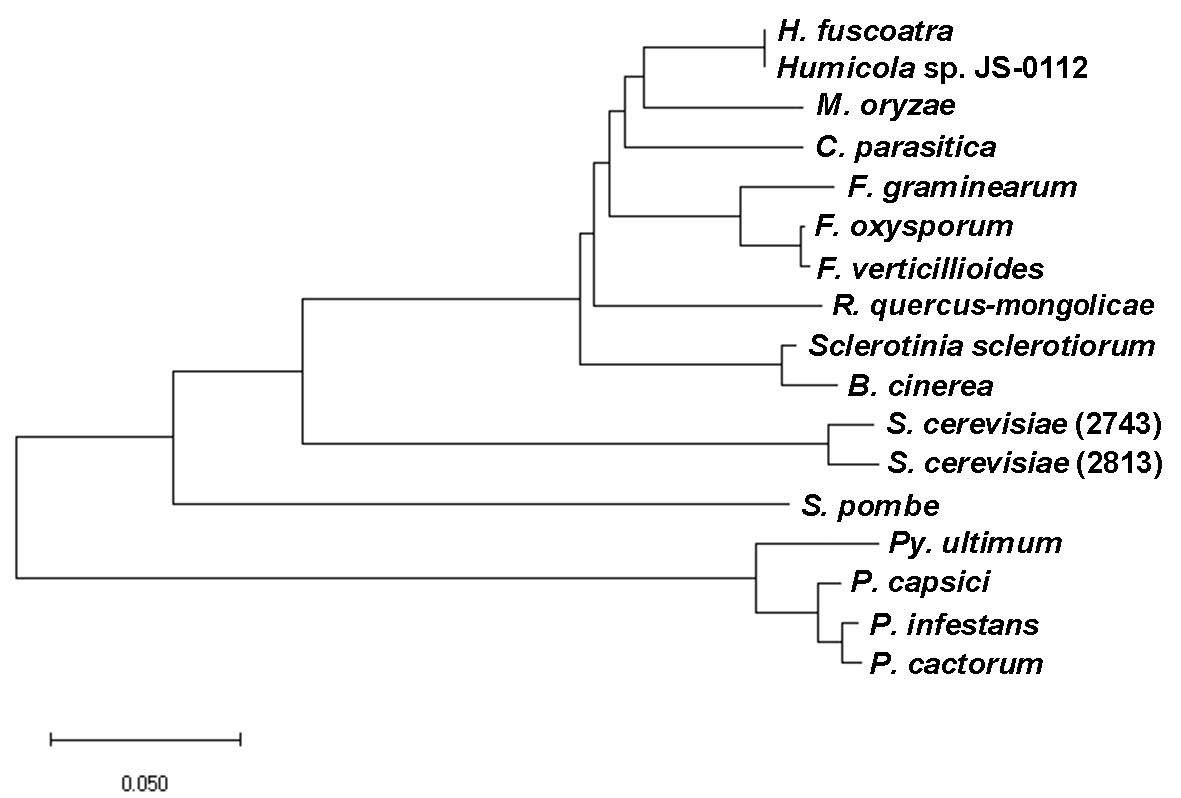


**Supplementary Figure S4 |** Phylogenetic tree of Hsp90 orthologs in fungi and oomycetes species. The alignment was performed with ClustalW, and the MEGA program Version 10.0 was used to perform a 1,000 bootstrap phylogenetic analysis using the neighbor joining method. Sequences were obtained from Comparative Fungal Genomics Platform (http://cfgp.riceblast.snu.ac.kr/).


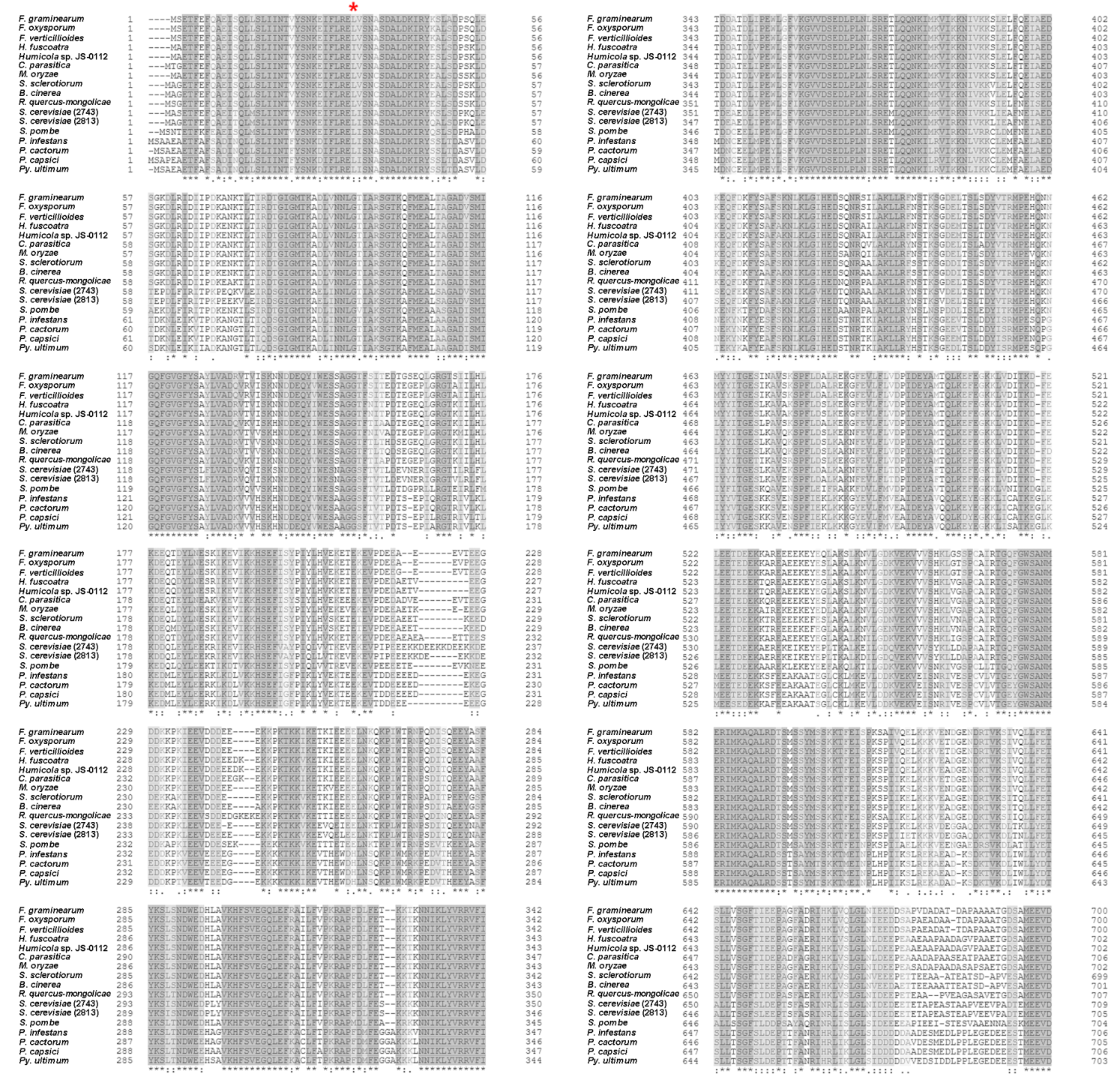


**Supplementary Figure S5 |** Alignment of amino acid sequences of Hsp90 orthologs from fungi and oomycetes. Alignment was performed according uning the Clustal Omega program available in the UniProt alignment tool (https://www.uniprot.org/align/). Common identical amino acid residues are shown in black background. Sequences were obtained from Comparative Fungal Genomics Platform (http://cfgp.riceblast.snu.ac.kr/).


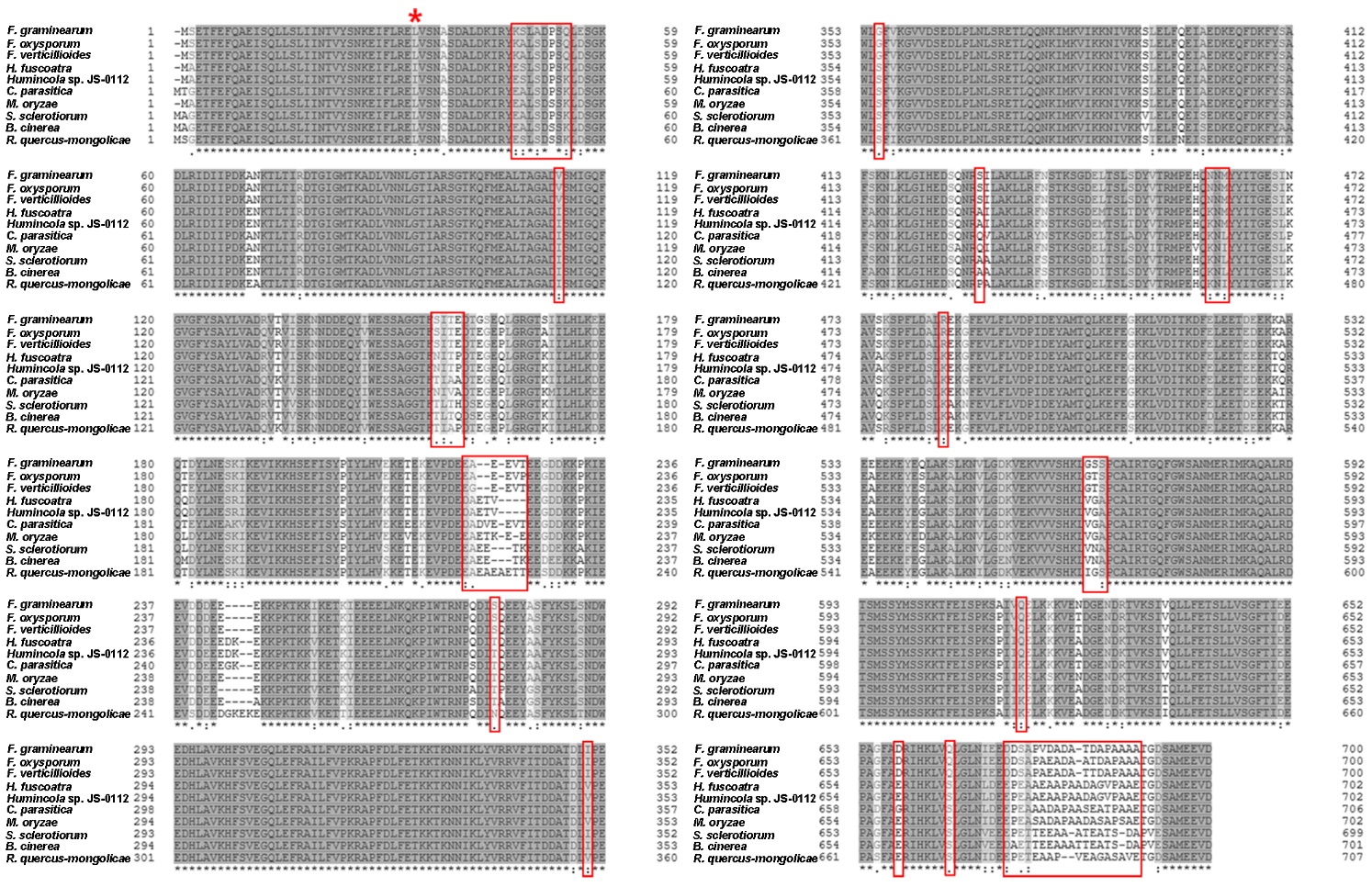


**Supplementary Figure S6 |** Alignment of amino acid sequences of Hsp90 orthologs from fungi species. Alignment was performed according uning the Clustal Omega program available in the UniProt alignment tool (https://www.uniprot.org/align/). Common identical amino acid residues are shown in black background and some of *Fusarium*-specific sequences were denoted as red box. Sequences were obtained from Comparative Fungal Genomics Platform (http://cfgp.riceblast.snu.ac.kr/).

**Supplementary Table S1** | List of fungi and oomycetes used in this study.

| Test fungus | Disease | Host and/or origin^*^ |
| --- | --- | --- |
| *Armillaria rolfsii* KACC40170 | Southern blight | Apple, KACC |
| *Botryosphaeria dothidea* KACC45481 | Apple botryosphaeria canker | Crab apple, KACC |
| *Botrytis cinerea* KACC40574 | Grey mold disease | Tomato, KACC |
| *Colletotrichum coccodes* KACC40010 | Anthracnose | Red pepper, KACC |
| *Cryphonectria parasitica* KACC40323 | Chestnut blight | Chestnut, KACC |
| *Fusarium graminearum* Z-3639 | Fusarium head blight | Wheat (Bowden & Leslie, 1999) |
| *Fusarium oxysporum* f.sp. *lycorpersici* | Fusarium wilt | Tomato, KRICT |
| *Magnaporthe oryzae* KACC46522 | Rice blast | Rice, KACC |
| *Ophiostoma ulmi* KACC40252 | Dutch elm disease | Elm, KACC |
| *Phytophthora cactorum* KACC40166 | Phytophthora stem rot | Apple, KACC |
| *Phytophthora cambivora* KACC40159 | Phytophthora collar rot | Apple, KACC |
| *Phytophthora capsici* KACC40158 | Phytophthora blight | Pepper, KACC |
| *Phytophthora cinnamomi* KACC40182 | Mating type, root rot, blight | Larch, KACC |
| *Phytophthora infestans* KACC40718 | Late blight | Potato, KACC |
| *Pythium graminicola* KACC40155 | Pythium blight | Creeping bentgrass, KACC |
| *Pythium helicoides* KACC42226 |  | Chrysanthemum compositae, KACC |
| *Pythium ultimum* KACC40705 | Damping off | Cucumber, KACC |
| *Raffaelea quercus-mongolicae* KACC44403 | Oak wilt | Oak, KACC |
| *Rhizoctonia solani* | Brown patch disease | Zoysia grass, DJ |
| *Sclerotinia homoeocarpa* | Dollar spot disease | Creeping bentgrass, DJ |
| *Taphrina wiesneri* KACC45487 | Witches’ broom | Oriental cherry, KACC |
| *Valsa kunzei* KACC44046 | Valsa canker | Apple, KACC |

^*KACC = Korean Agriculture collection culture, KRICT = Korean Research Institute of Chemical Technology, DJ=Daejeong-Golf Engineering Co. Ltd.^

**Supplementary Table S2 |** *F. graminearum* strains used in this study.

| **Strain** | **Genotype** | **Reference, source, or parent strains** |
| --- | --- | --- |
| Z-3639 | *Fusarium graminearum* wild-type | Bowden & Leslie, 1999 |
| Δ*mat2* | Δ*mat1-2::GFP-HYG* | Lee *et al*, 2003 |
| HK226 (*FgHSP90:: P_ZEAR_-FgHSP90*) | *FgHSP90::HYG-P_ZEAR_-FgHSP90* | Bui *et al*, 2016 |
| *P_EF1α_-CpHSP90* | *GEN-P_EF1α_-CpHSP90* | Z-3639 |
| Δ*mat2*; *P_EF1α_-CpHSP90* | Δ*mat1-2::GFP-HYG*; *GEN-P_EF1α_-CpHSP90* | Δ*mat2* × *P_EF1α_-CpHSP90* |
| *FgHSP90:: P_ZEAR_-FgHSP90*; *P_EF1α_-CpHSP90* | *FgHSP90::P_ZEAR_-FgHSP90;P_EF1α_-CpHSP90* | Δ*mat2*; *P_EF1α_-CpHSP90* × *P_EF1α_-CpHSP90* |

**Supplementary Table** **S3** | Preliminary screening of endophytic fungi from plant for antifungal activity against *Sclerotinia homoeocarpa*.

| No | Code No | Scientific name | Plant origin | | MIC^a^ value against *S. homoeocarpa* | |
| --- | --- | --- | --- | --- | --- | --- |
|  |  |  | Scientific name  (Collection site) | Tissue | Culture filtrate (%) | Mycelial extract (µg mL^−1^) |
| 1 | JS - 0001 | *Colletotrichum boninense* | *Cephalotaxus harringtonia* (Wando, Chonnam) | leaf | - | - |
| 2 | JS - 0002 | *Colletotrichum boninense* | *Cephalotaxus harringtonia* (Wando, Chonnam) | leaf | - | - |
| 3 | JS - 0004 | *Phyllosticta pyrolae* | *Cephalotaxus harringtonia* (Wando, Chonnam) | leaf | - | - |
| 4 | JS - 0020 | *Phomopsis* sp*.* | *Euonymus japonicas* (Wando, Chonnam) | leaf | - | - |
| 5 | JS - 0042 | *Glomerella acutata* | *Ulmus davidiana* var. *japonica* (Moongyeong, Gyeongbuk) | stem | - | - |
| 6 | JS - 0043 | *Aureobasidium pullulans* | *Ulmus davidiana* var. *japonica* (Moongyeong, Gyeongbuk) | stem | - | - |
| 7 | JS - 0044 | *Phomopsis* sp*.* | *Ulmus davidiana* var. *japonica* (Moongyeong, Gyeongbuk) | stem | - | - |
| 8 | JS - 0045 | *Diaporthe* sp*.* | *Ulmus davidiana* var. *japonica* (Moongyeong, Gyeongbuk) | stem | - | - |
| 9 | JS - 0046 | *Diaporthe* sp*.* | *Ulmus davidiana* var. *japonicab* (Moongyeong, Gyeongbuk) | stem | - | - |
| 10 | JS - 0047 | *Diaporthe* sp*.* | *Ulmus davidiana* var. *japonica* | stem | - | - |
| 11 | JS - 0048 | *Phomopsis* sp*.* | *Ulmus davidiana* var. *japonica* (Moongyeong, Gyeongbuk) | leaf | - | - |
| 12 | JS - 0061 | *Diaporthe eres* | *Morus alba* (Choongju, Choongbuk) | stem | - | - |
| 13 | JS - 0062 | *Alternaria alternata* | *Morus alba* (Choongju, Choongbuk) | stem | - | - |
| 14 | JS - 0064 | *Alternaria alternata* | *Morus alba* (Choongju, Choongbuk) | stem | - | - |
| 15 | JS - 0065 | *Phomopsis longicolla* | *Morus alba* (Choongju, Choongbuk) | stem | - | - |
| 16 | JS - 0066 | *Phomopsis longicolla* | *Morus alba* (Choongju, Choongbuk) | stem | - | - |
| 17 | JS - 0067 | *Cladosporium* sp*.* | *Morus alba* (Moongyeong, Gyeongbuk) | stem | - | - |
| 18 | JS - 0112 | *Humicola* sp*.* | *Ixeris repens* (Silmi-do, Incheon) | root | 1.25 | 125 |
| 19 | JS - 0169 | *Fusarium solani* | *Morus alba* (Choongju, Choongbuk) | leaf | >20 | - |
| 20 | JS - 0170 | *Fusarium solani* | *Morus alba* (Choongju, Choongbuk) | leaf | - | - |
| 21 | JS - 0171 | *Phomopsis* sp*.* | *Morus alba* (Choongju, Choongbuk) | stem | - | - |
| 22 | JS - 0174 | *Fusarium solani* | *Morus alba* (Choongju, Choongbuk) | stem | - | - |
| 23 | JS - 0175 | *Aschersonia* sp*.* | *Morus alba* (Choongju, Choongbuk) | stem | - | - |
| 24 | JS - 0176 | *Phoma* sp*.* | *Morus alba* (Moongyeong, Gyeongbuk) | leaf | - | - |
| 25 | JS - 0228 | *Phoma sp.* | *Morus alba* (Inje, Gangwon) | leaf | - | - |
| 26 | JS - 0229 | *Alternaria alternata* | *Morus alba* (Inje, Gangwon) | stem | - | - |
| 27 | JS - 0230 | *Paraconiothyrium* sp*.* | *Morus alba* (Inje, Gangwon) | stem | - | - |
| 28 | JS - 0255 | *Colletotrichum boninense* | *Morus alba* (Yangyang, Gangwon) | stem | - | - |
| 29 | JS - 0284 | *Phomopsis* sp*.* | *Morus alba* (Pyeongchang, Gangwon) | stem | - | - |
| 30 | JS - 0358 | *Glomerella acutata* | *Morus alba* (Inje, Gangwon) | leaf | - | - |
| 31 | JS - 0360 | *Phoma herbarum* | *Morus alba* (Inje, Gangwon) | leaf | - | - |
| 32 | JS - 0361 | *Colletotrichum nymphaeae* | *Morus alba* (Inje, Gangwon) | leaf | - | - |
| 33 | JS - 0369 | *Glomerella cingulata* | *Morus alba* (Inje, Gangwon) | stem | - | - |
| 34 | JS - 0432 | *Diaporthe helianthi* | *Plantago major* f. *jezomariima* (Mo-do, Incheon) | leaf | - | - |
| 35 | JS - 0433 | *Diaporthe phaseolorum* | *Plantago major* f. *jezomariima* (Mo-do, Incheon) | leaf | - | - |
| 36 | JS - 0434 | *Colletotrichum gloeosporioides* | *Plantago major* f. *jezomariima* (Mo-do, Incheon) | leaf | - | - |
| 37 | JS - 0435 | *Alternaria arborescens* | *Plantago major* f. *jezomariima* (Mo-do, Incheon) | leaf | - | - |
| 38 | JS - 0436 | *Colletotrichum gloeosporioides* | *Plantago major* f. *jezomariima* (Mo-do, Incheon) | stem | - | - |
| 39 | JS - 0437 | *Phomopsis* sp*.* | *Plantago major* f. *jezomariima* (Mo-do, Incheon) | root | - | - |
| 40 | JS - 0439 | *Phomopsis liquidambari* | *Plantago major* f. *jezomariima* (Mo-do, Incheon) | root | - | - |
| 41 | JS - 0440 | *Guignardia mangiferae* | *Plantago major* f. *jezomariima* (Mo-do, Incheon) | root | - | - |
| 42 | JS - 0441 | *Fusarium oxysporum* | *Plantago major* f. *jezomariima* (Mo-do, Incheon) | root | - | - |
| 43 | JS - 0496 | *Alternaria alternata* | *Phytolacca Americana* (Taean, Choongnam) | stem | - | - |
| 44 | JS - 0497 | *Fusarium lateritium* | *Phytolacca Americana* (Taean, Choongnam) | fruit | - | - |
| 45 | JS - 0595 | *Cladosporium* sp*.* | *Davallia mariesii* (Goolup-do, Incheon) | root | - | - |
| 46 | JS - 0596 | *Pestalotiopsis* sp*.* | *Davallia mariesii* (Goolup-do, Incheon) | root | - | - |
| 47 | JS - 0611 | *Alternaria tenuissima* | *Ixeris repens* (Goolup-do, Incheon) | stem | - | - |
| 48 | JS - 0613 | *Alternaria tenuissima* | *Ixeris repens* (Goolup-do, Incheon) | leaf | - | - |
| 49 | JS - 0614 | *Fusarium armeniacum* | *Ixeris repens* (Goolup-do, Incheon) | leaf | - | - |
| 50 | JS - 0639 | *Fusarium acuminatum* | *Illicium anisatum* (Goolup-do, Incheon) | leaf | - | - |
| 51 | JS - 0783 | *Fusarium lateritium* | *Korthalsella japonica* (Geomoon-do, Chonnam) | stem | - | - |
| 52 | JS - 0785 | *Glomerella fioriniae* | *Korthalsella japonica* (Geomoon-do, Chonnam) | leaf | - | - |
| 53 | JS - 0786 | *Glomerella fioriniae* | *Korthalsella japonica* (Geomoon-do, Chonnam) | leaf | - | - |
| 54 | JS - 0845 | *Alternaria* sp*.* | *Cudrania tricuspidata* (Seocheon, Choongnam) | stem | - | - |
| 55 | JS - 0847 | *Alternaria* sp*.* | *Cudrania tricuspidata* (Seocheon, Choongnam) | stem | - | - |
| 56 | JS - 0877 | *Glomerella fioriniae* | *Zanthoxylum schinifolium* (Seogwipo, Jeju) | leaf | - | - |
| 57 | JS - 0878 | *Alternaria* sp*.* | *Zanthoxylum schinifolium* (Seogwipo, Jeju) | leaf | - | - |
| 58 | JS - 0879 | *Phomopsis* sp*.* | *Zanthoxylum schinifolium* (Seogwipo, Jeju) | leaf | - | - |
| 59 | JS - 0894 | *Cladosporium oxysporum* | *Abies koreana* (Seogwipo, Jeju) | stem | - | - |
| 60 | JS - 0895 | *Helotiales* sp*.* | *Abies koreana* (Seogwipo, Jeju) | stem | - | - |
| 61 | JS - 0932 | *Fusarium* sp*.* | *Euonymus fortunei* var. *radicans* (Jeju, Jeju) | stem | - | - |
| 62 | JS - 0933 | *Glomerella fioriniae* | *Euonymus fortunei* var. *radicans* (Jeju, Jeju) | stem | - | - |
| 63 | JS - 0935 | *Neofabraea malicorticis* | *Euonymus fortunei* var. *radicans* (Jeju, Jeju) | leaf | - | - |
| 64 | JS - 0960 | *Alternaria alternata* | *Taxus cuspidate* (Pyeongchang, Gangwon) | stem | - | - |
| 65 | JS - 0962 | *Lophiostoma* sp*.* | *Taxus cuspidate* (Pyeongchang, Gangwon) | leaf | - | - |
| 66 | JS - 0971 | *Neonectria veuillotiana* | *Taxus cuspidate* (Muju, Chonbuk) | stem | - | - |
| 67 | JS - 0972 | *Aspergillus flavus* | *Taxus cuspidate* (Muju, Chonbuk) | stem | - | - |
| 68 | JS - 0973 | *Gelasinospora brevispora* | *Taxus cuspidate* (Muju, Chonbuk) | stem | - | - |
| 69 | JS - 0974 | *Pestalotiopsis* sp*.* | *Taxus cuspidate* (Muju, Chonbuk) | stem | - | - |
| 70 | JS - 0975 | *Aureobasidium* sp*.* | *Taxus cuspidate* (Muju, Chonbuk) | stem | - | - |
| 71 | JS - 0977 | *Aspergillus fumigatus* | *Taxus cuspidate* (Muju, Chonbuk) | stem | - | - |
| 72 | JS - 0978 | *Neonectria galligena* | *Taxus cuspidate* (Muju, Chonbuk) | stem | - | - |
| 73 | JS - 0979 | *Glomerella fioriniae* | *Taxus cuspidate* (Muju, Chonbuk) | stem | - | - |
| 74 | JS - 0982 | *Pestalotiopsis* sp*.* | *Taxus cuspidate* (Muju, Chonbuk) | stem | - | - |
| 75 | JS - 0983 | *Davidiella* sp*.* | *Taxus cuspidate* (Muju, Chonbuk) | stem | - | - |
| 76 | JS - 1043 | *Helminthosporium* sp*.* | *Torreya nucifera* (Jeju, Jeju) | stem | - | - |
| 77 | JS - 1067 | *Pestalotiopsis microspora* | *Eriobotrya japonica* (Seogwipo, Jeju) | stem | - | - |
| 78 | JS - 1068 | *Glomerella fioriniae* | *Eriobotrya japonica* (Seogwipo, Jeju) | stem | - | - |
| 79 | JS - 1069 | *Glomerella fioriniae* | *Eriobotrya japonica* (Seogwipo, Jeju) | stem | - | - |
| 80 | JS - 1071 | *Pestalotiopsis microspora* | *Eriobotrya japonica* (Seogwipo, Jeju) | stem | - | - |
| 81 | JS - 1072 | *Glomerella fioriniae* | *Eriobotrya japonica* (Seogwipo, Jeju) | leaf | - | - |
| 82 | JS - 1073 | *Glomerella fioriniae* | *Eriobotrya japonica* (Seogwipo, Jeju) | leaf | - | - |
| 83 | JS - 1075 | *Glomerella fioriniae* | *Eriobotrya japonica* (Seogwipo, Jeju) | leaf | - | - |
| 84 | JS - 1170 | *Pestalotiopsis* sp*.* | *Taxus cuspidate* (Ulleung-do, Gyeongbuk) | stem | - | - |
| 85 | JS - 1171 | *Bionectria ochroleuca* | *Taxus cuspidate* (Ulleung-do, Gyeongbuk) | stem | - | - |
| 86 | JS - 1172 | *Glomerella fioriniae* | *Taxus cuspidate* (Ulleung-do, Gyeongbuk) | leaf | - | - |
| 87 | JS - 1173 | *Colletotrichum aenigma* | *Taxus cuspidate* (Ulleung-do, Gyeongbuk) | leaf | - | - |
| 88 | JS - 1250 | *Coniothyrium* sp*.* | *Abies nephrolepis* (Jungsun, Gangwon) | stem | - | - |
| 89 | JS - 1252 | *Fusarium cortaderiae* | *Abies nephrolepis* (Ulleung-do, Gyeongbuk) | leaf | - | - |
| 90 | JS - 1263 | *Pestalotiopsis* sp*.* | *Dryopteris pacifica* (Yeosu, Chonnam) | stem | - | - |
| 91 | JS - 1265 | *Hypocrea lixii* | *Abies nephrolepis* (Taebaek, Gangwon) | stem | - | - |
| 92 | JS - 1283 | *Alternaria alternata* | *Abies holophylla* (Seolak, Gangwon) | stem | - | - |
| 93 | JS - 1285 | *Phoma* sp*.* | *Abies holophylla* (Seolak, Gangwon) | stem | - | - |
| 94 | JS - 1286 | *Amphirosellinia nigrospora* | *Abies holophylla* (Seolak, Gangwon) | stem | - | - |
| 95 | JS - 1287 | *Biscogniauxia maritima* | *Abies holophylla* (Seolak, Gangwon) | leaf | - | - |
| 96 | JS - 1291 | *Microdiplodia* sp*.* | *Abies koreana* (Muju, Chonbuk) | stem | - | - |
| 97 | JS - 1293 | *Periconia macrospinosa* | *Abies koreana* (Muju, Chonbuk) | stem | - | - |
| 98 | JS - 1297 | *Arthrinium arundinis* | *Abies holophylla* (Muju, Chonbuk) | leaf | - | - |
| 99 | JS - 1303 | *Dicarpella dryina* | *Abies koreana* (Muju, Chonbuk) | stem | - | - |
| 100 | JS - 1309 | *Phomopsis* sp*.* | *Abies koreana* (Muju, Chonbuk) | leaf | - | - |
| 101 | JS - 1311 | *Hypocrea alni* | *Abies koreana* (Muju, Chonbuk) | stem | - | - |
| 102 | JS - 1312 | *Hypocrea lixii* | *Abies koreana* (Muju, Chonbuk) | stem | - | - |
| 103 | JS - 1316 | *Diaporthe* sp*.* | *Abies koreana* (Muju, Chonbuk) | leaf | - | - |
| 104 | JS - 1317 | *Phomopsis* sp*.* | *Abies koreana* (Muju, Chonbuk) | leaf | - | - |
| 105 | JS - 1320 | *Fusarium ciliatum* | *Abies holophylla* (Seolak, Gangwon) | stem | - | - |
| 106 | JS - 1324 | *Coniochaeta* sp*.* | *Abies koreana* (Muju, Chonbuk) | stem | - | - |
| 107 | JS - 1326 | *Phanerochaete sordida* | *Abies koreana* (Muju, Chonbuk) | stem | - | - |
| 108 | JS - 1330 | *Fusarium ciliatum* | *Abies koreana* (Muju, Chonbuk) | stem | - | - |
| 109 | JS - 1334 | *Phomopsis* sp*.* | *Abies koreana* (Muju, Chonbuk) | leaf | - | - |
| 110 | JS - 1337 | *Nigrospora* sp*.* | *Abies koreana* (Gwangyang, Chonnam) | leaf | - | - |
| 111 | JS - 1339 | *Dicarpella dryina* | *Abies nephrolepis* (Danyang, Choongbuk) | stem | - | - |
| 112 | JS - 1344 | *Alternaria brassicicola* | *Abies nephrolepis* (Danyang, Choongbuk) | leaf | - | - |
| 113 | JS - 1345 | *Daldinia childiae* | *Abies koreana* (Hwasoon, Chonnam) | stem | - | - |
| 114 | JS - 1346 | *Diaporthe* sp*.* | *Abies koreana* (Hwasoon, Chonnam) | stem | - | - |
| 115 | JS - 1347 | *Nemania diffusa* | *Abies koreana* (Hwasoon, Chonnam) | stem | - | - |
| 116 | JS - 1348 | *Diaporthe eres* | *Abies koreana* (Hwasoon, Chonnam) | stem | - | - |
| 117 | JS - 1349 | *Diaporthe* sp*.* | *Abies koreana* (Hwasoon, Chonnam) | stem | - | - |
| 118 | JS - 1352 | *Diaporthe* sp*.* | *Abies koreana* (Hwasoon, Chonnam) | stem | - | - |
| 119 | JS - 1353 | *Diaporthe* sp*.* | *Abies koreana* (Hwasoon, Chonnam) | stem | - | - |
| 120 | JS - 1356 | *Alternaria brassicicola* | *Abies koreana* (Hwasoon, Chonnam) | stem | - | - |
| 121 | JS - 1358 | *Alternaria* sp*.* | *Abies koreana* (Hwasoon, Chonnam) | stem | - | - |
| 122 | JS - 1367 | *Mollisia fusca* | *Abies koreana* (Hwasoon, Chonnam) | stem | - | - |
| 123 | JS - 1368 | *Mycosphaerella* sp*.* | *Abies koreana* (Hwasoon, Chonnam) | stem | - | - |
| 124 | JS - 1370 | *Diaporthe* sp. | *Abies koreana* (Hwasoon, Chonnam) | stem | - | - |
| 125 | JS - 1372 | *Dictyochaeta simplex* | *Polystichum tsus-simense* (Seogwipo, Jeju) | leaf | - | - |
| 126 | JS - 1373 | *Nectria rigidiuscula* | *Polystichum tsus-simense* (Seogwipo, Jeju) | leaf | - | - |
| 127 | JS - 1374 | *Fusarium* sp*.* | *Polystichum tsus-simense* (Seogwipo, Jeju) | leaf | - | - |
| 128 | JS - 1378 | *Pestalotiopsis* sp*.* | *Polystichum tsus-simense* (Seogwipo, Jeju) | leaf | - | - |
| 129 | JS - 1384 | *Neofusicoccum mangiferae* | *Polystichum lepidocaulon* (Seogwipo, Jeju) | stem | - | - |
| 130 | JS - 1386 | *Pestalotiopsis* sp*.* | *Polystichum lepidocaulon* (Seogwipo, Jeju) | leaf | - | - |
| 131 | JS - 1387 | *Pestalotiopsis protearum* | *Polystichum lepidocaulon* (Seogwipo, Jeju) | stem | - | - |
| 132 | JS - 1395 | *Gibberella* sp*.* | *Polystichum lepidocaulon* (Seogwipo, Jeju) | leaf | - | - |
| 133 | JS - 1396 | *Fusarium solani* | *Polystichum lepidocaulon* (Seogwipo, Jeju) | stem | - | - |
| 134 | JS - 1398 | *Gibberella* sp*.* | *Polystichum lepidocaulon* (Seogwipo, Jeju) | leaf | - | - |
| 135 | JS - 1399 | *Glomerella fioriniae* | *Polystichum lepidocaulon* (Seogwipo, Jeju) | leaf | - | - |
| 136 | JS - 1400 | *Alternaria* sp*.* | *Polystichum lepidocaulon* (Seogwipo, Jeju) | stem | - | - |
| 137 | JS - 1402 | *Lasiodiplodia pseudotheobromae* | *Polystichum lepidocaulon* (Seogwipo, Jeju) | stem | - | - |
| 138 | JS - 1413 | *Alternaria* sp*.* | *Pteris cretica* (Seogwipo, Jeju) | stem | - | - |
| 139 | JS - 1414 | *Chaetomium* sp*.* | *Pteris cretica* (Seogwipo, Jeju) | stem | - | - |
| 140 | JS - 1415 | *Pestalotiopsis protearum* | *Pteris cretica* (Seogwipo, Jeju) | leaf | - | - |
| 141 | JS - 1416 | *Nemania diffusa* | *Pteris cretica* (Seogwipo, Jeju) | leaf | - | - |
| 142 | JS - 1418 | *Colletotrichum aenigma* | *Dryopteris erythrosora* (Seogwipo, Jeju) | stem | - | - |
| 143 | JS - 1419 | *Amphirosellinia nigrospora* | *Dryopteris erythrosora* (Seogwipo, Jeju) | stem | - | - |
| 144 | JS - 1420 | *Glomerella fioriniae* | *Dryopteris erythrosora* (Seogwipo, Jeju) | stem | - | - |
| 145 | JS - 1421 | *Bionectria ochroleuca* | *Dryopteris erythrosora* (Seogwipo, Jeju) | stem | - | - |
| 146 | JS - 1423 | *Colletotrichum gloeosporioides* | *Dryopteris erythrosora* (Seogwipo, Jeju) | leaf | - | - |
| 147 | JS - 1439 | *Alternaria arborescens* | *Cyclosorus acuminatus* (Seogwipo, Jeju) | stem | - | - |
| 148 | JS - 1440 | *Gibellulopsis nigrescens* | *Cyclosorus acuminatus* (Seogwipo, Jeju) | leaf | - | - |
| 149 | JS - 1441 | *Glomerella fioriniae* | *Cyclosorus acuminatus* (Seogwipo, Jeju) | leaf | - | - |
| 150 | JS - 1442 | *Colletotrichum boninense* | *Cyclosorus acuminatus* (Seogwipo, Jeju) | leaf | - | - |
| 151 | JS - 1455 | *Alternaria* sp*.* | *Cyrtomium falcatum* (Seogwipo, Jeju) | stem | - | - |
| 152 | JS - 1456 | *Colletotrichum aenigma* | *Cyrtomium falcatum* (Seogwipo, Jeju) | leaf | - | - |
| 153 | JS - 1458 | *Lasiodiplodia pseudotheobromae* | *Coniogramme intermedia* (Seogwipo, Jeju) | stem | - | - |
| 154 | JS - 1459 | *Nigrospora* sp*.* | *Coniogramme intermedia* (Seogwipo, Jeju) | leaf | - | - |
| 155 | JS - 1463 | *Colletotrichum boninense* | *Dryopteris bissetiana* (Seogwipo, Jeju) | stem | - | - |
| 156 | JS - 1465 | *Pestalotiopsis protearum* | *Dryopteris bissetiana* (Seogwipo, Jeju) | leaf | - | - |
| 157 | JS - 1466 | *Phaeosphaeriopsis* sp*.* | *Dryopteris bissetiana* (Seogwipo, Jeju) | leaf | - | - |
| 158 | JS - 1467 | *Fusarium* sp*.* | *Dryopteris bissetiana* (Seogwipo, Jeju) | leaf | - | - |
| 159 | JS - 1468 | *Pestalotiopsis jesteri* | *Dryopteris bissetiana* (Seogwipo, Jeju) | leaf | - | - |
| 160 | JS - 1483 | *Colletotrichum aenigma* | *Dryopteris bissetiana* (Seogwipo, Jeju) | stem | - | - |
| 161 | JS - 1485 | *Nemania diffusa* | *Dryopteris bissetiana* (Seogwipo, Jeju) | stem | - | - |
| 162 | JS - 1486 | *Colletotrichum aenigma* | *Dryopteris bissetiana* (Seogwipo, Jeju) | leaf | - | - |
| 163 | JS - 1488 | *Alternaria arborescens* | *Torreya nucifera* (Seogwipo, Jeju) | stem | - | - |
| 164 | JS - 1491 | *Nemania diffusa* | *Torreya nucifera* (Seogwipo, Jeju) | leaf | - | - |
| 165 | JS - 1493 | *Nemania diffusa* | *Torreya nucifera* (Seogwipo, Jeju) | leaf | - | - |
| 166 | JS - 1494 | *Pestalotiopsis* sp*.* | *Dryopteris varia* (Seogwipo, Jeju) | stem | - | - |
| 167 | JS - 1495 | *Colletotrichum aenigma* | *Dryopteris varia* (Seogwipo, Jeju) | stem | - | - |
| 168 | JS - 1496 | *Colletotrichum aenigma* | *Dryopteris varia* (Seogwipo, Jeju) | leaf | - | - |
| 169 | JS - 1497 | *Colletotrichum aenigma* | *Dryopteris varia* (Seogwipo, Jeju) | leaf | - | - |
| 170 | JS - 1575 | *Colletotrichum aenigma* | *Euonymus japonicas* (Seogwipo, Jeju) | leaf | - | - |
| 171 | JS - 1576 | *Colletotrichum* sp*.* | *Euonymus japonicas* (Seogwipo, Jeju) | leaf | - | - |
| 172 | JS - 1583 | *Halorosellinia* sp*.* | *Dryopteris bissetiana* (Seogwipo, Jeju) | stem | - | - |
| 173 | JS - 1585 | *Alternaria* sp*.* | *Dryopteris varia* (Seogwipo, Jeju) | stem | - | - |
| 174 | JS - 1604 | *Alternaria* sp*.* | *Abies koreana* (Hapcheon, Gyeongnam) | stem | - | - |
| 175 | JS - 1605 | *Celosporium larixicola* | *Abies koreana* (Hapcheon, Gyeongnam) | stem | - | - |
| 176 | JS - 1606 | *Alternaria tenuissima* | *Abies koreana* (Hapcheon, Gyeongnam) | leaf | - | - |
| 177 | JS - 1608 | *Hormonema* sp*.* | *Abies koreana* (Hapcheon, Gyeongnam) | stem | - | - |
| 178 | JS - 1612 | *Colletotrichum aenigma* | *Abies koreana* (Hapcheon, Gyeongnam) | stem | - | - |
| 179 | JS - 1614 | *Colletotrichum kahawae* | *Abies koreana* (Sungju, Gyeongnam) | stem | - | - |
| 180 | JS - 1615 | *Alternaria* sp. | *Abies koreana* (Sungju, Gyeongnam) | stem | - | - |
| 181 | JS - 1616 | *Alternaria* sp*.* | *Abies koreana* (Sungju, Gyeongnam) | leaf | - | - |
| 182 | JS - 1618 | *Alternaria* sp*.* | *Abies koreana* (Sungju, Gyeongnam) | stem | - | - |
| 183 | JS - 1623 | *Fusarium* sp*.* | *Abies koreana* (Sungju, Gyeongnam) | stem | - | - |
| 184 | JS - 1626 | *Alternaria* sp*.* | *Abies koreana* (Sungju, Gyeongnam) | leaf | - | - |
| 185 | JS - 1632 | *Alternaria* sp*.* | *Abies koreana* (Sungju, Gyeongnam) | stem | - | - |
| 186 | JS - 1633 | *Phialocephala fortinii* | *Abies koreana* (Sancheong, Gyeongnam) | stem | - | - |
| 187 | JS - 1634 | *Nemania* sp*.* | *Abies koreana* (Sancheong, Gyeongnam) | stem | - | - |
| 188 | JS - 1635 | *Aschersonia* sp*.* | *Abies koreana* (Sancheong, Gyeongnam) | stem | - | - |
| 189 | JS - 1638 | *Pestalotiopsis* sp*.* | *Abies koreana* (Sancheong, Gyeongnam) | stem | - | - |
| 190 | JS - 1639 | *Fusarium* sp*.* | *Abies koreana* (Sancheong, Gyeongnam) | stem | - | - |
| 191 | JS - 1642 | *Hormonema* sp*.* | *Abies koreana* (Sancheong, Gyeongnam) | stem | - | - |
| 192 | JS - 1643 | *Phialocephala fortinii* | *Abies koreana* (Sancheong, Gyeongnam) | leaf | - | - |
| 193 | JS - 1644 | *Alternaria alternata* | *Abies koreana* (Sancheong, Gyeongnam) | stem | - | - |
| 194 | JS - 1647 | *Phaeosphaeriopsis* sp*.* | *Abies koreana* (Sancheong, Gyeongnam) | stem | - | - |
| 195 | JS - 1650 | *Colletotrichum aenigma* | *Abies koreana* (Sancheong, Gyeongnam) | stem | - | - |
| 196 | JS - 1651 | *Colletotrichum aenigma* | *Abies koreana* (Sancheong, Gyeongnam) | stem | - | - |
| 197 | JS - 1652 | *Hormonema* sp*.* | *Abies koreana* (Sancheong, Gyeongnam) | stem | - | - |
| 198 | JS - 1654 | *Alternaria* sp*.* | *Abies koreana* (Hamyang, Gyeongnam) | stem | - | - |
| 199 | JS - 1655 | *Nemania* sp*.* | *Abies koreana* (Hamyang, Gyeongnam) | stem | - | - |
| 200 | JS - 1656 | *Helminthosporium velutinum* | *Abies koreana* (Sancheong, Gyeongnam) | stem | - | - |
| 201 | JS - 1658 | *Hormonema* sp*.* | *Abies koreana* (Sancheong, Gyeongnam) | stem | - | - |
| 202 | JS - 1660 | *Mollisia fusca* | *Abies koreana* (Sancheong, Gyeongnam) | stem | - | - |
| 203 | JS - 1661 | *Phialocephala* sp*.* | *Abies koreana* (Sancheong, Gyeongnam) | stem | - | - |
| 204 | JS - 1662 | *Nigrospora* sp*.* | *Abies koreana* (Sancheong, Gyeongnam) | stem | - | - |
| 205 | JS - 1664 | *Hormonema* sp*.* | *Abies koreana* (Sancheong, Gyeongnam) | stem | - | - |
| 206 | JS - 1665 | *Hormonema* sp*.* | *Abies koreana* (Sancheong, Gyeongnam) | stem | - | - |
| 207 | JS - 1666 | *Epicoccum nigrum* | *Abies koreana* (Sancheong, Gyeongnam) | stem | - | - |
| 208 | JS - 1668 | *Nemania* sp*.* | *Abies koreana* (Sancheong, Gyeongnam) | stem | - | - |
| 209 | JS - 1669 | *Hormonema* sp*.* | *Abies koreana* (Sancheong, Gyeongnam) | stem | - | - |
| 210 | JS - 1670 | *Diaporthe* sp*.* | *Abies koreana* (Sancheong, Gyeongnam) | stem | - | - |
| 211 | JS - 1676 | *Nemania diffusa* | *Dryopteris erythrosora* (Seogwipo, Jeju) | stem | - | - |
| 212 | JS - 1682 | *Monochaetia* sp*.* | *Dryopteris bissetiana* (Seogwipo, Jeju) | stem | - | - |
| 213 | JS - 1706 | *Pestalotiopsis* sp*.* | *Torreya nucifera* (Jeju, Jeju) | leaf | - | - |
| 214 | JS - 1707 | *Fusarium lateritium* | *Torreya nucifera* (Jeju, Jeju) | leaf | - | - |
| 215 | JS - 1709 | *Glomerella fioriniae* | *Torreya nucifera* (Jeju, Jeju) | stem | - | - |
| 216 | JS - 1712 | *Colletotrichum aenigma* | *Torreya nucifera* (Jeju, Jeju) | stem | - | - |
| 217 | JS - 1729 | *Nemania* sp*.* | *Abies koreana* (Seogwipo, Jeju) | stem | - | - |
| 218 | JS - 1731 | *Nemania diffusa* | *Abies koreana* (Seogwipo, Jeju) | stem | - | - |
| 219 | JS - 1733 | *Nodulisporium* sp*.* | *Abies koreana* (Seogwipo, Jeju) | stem | - | - |

-: No inhibition was detected

^a^ MIC: minimum inhibitory concentration. All data were obtained from three replicates.

**Supplementary Table S4** | Primers used in this study for generation of transgenic *F. graminearum* strains.

| **Primer** | **Sequence (5′ → 3′)** |
| --- | --- |
| pSKGEN-A | CGACGGCCAGTGAATTGTAATACGACTC |
| pSKGEN-B | GGGGCGTCGGTTTCCACTATC |
| pSKGEN-C | gaactcagaaacgcgacggctCTTTGAAGATTGGGTTCCTTTTGTG |
| Cp-A | cacaaaaggaacccaatcttcaaagAGCCGTCGCGTTTCTGAGTTC |
| Cp-B | CTGTGAACCCGAGCTCCGTATTA |
| Cp-C | ACCCGTACCAAACTCCTGAACCA |
| H3 | CGTTATGTTTATCGGCACTTTGC |
| ZEAR-F3 | GATGTAGGAGGGCGTGGATATGT |

**Supplementary Table S5 |** ^1^H and ^13^C-NMR spectra of monorden isolated from *Humicola* sp. JS-0112.

| Position | Monorden | |
| --- | --- | --- |
|  | ^13^C^a^ | ^1^H (multi., *J*) |
| 1 |  |  |
| 2 | 72.22 | 5.38 (1H, m) |
| 3 | 37.82 | 2.41 (1Ha, dt, J = 3.4, 14.7 Hz); 1.70 (1Hb, m) |
| 4 | 56.95 | 3.32 (1H, br, s) |
| 5 | 56.60 | 3.06 (1H, br, dt, J = 2.0, 10.0 Hz) |
| 6 | 130.98 | 5.77 (1H, dd, J = 4.0, 10.8 Hz) |
| 7 | 135.18 | 6.20 (1H, dd) |
| 8 | 140.83 | 7.59 (1H, dd, J = 9.9, 16.1 Hz) |
| 9 | 131.62 | 6.08 (1H, dd, J = 16.1 Hz) |
| 10 | 199.69 |  |
| 11 | 46.58 | 4.14 (1Ha, d, J = 16.3 Hz); 3.92 (1Hb, d, J = 16.3 Hz,) |
| 12 | 137.16 |  |
| 13 | 115.18 |  |
| 14 | 159.06 |  |
| 15 | 103.87 | 6.64 (1H, s) |
| 16 | 158.03 |  |
| 17 | 113.64 |  |
| 18 | 168.15 |  |
| 19 | 18.84 | 1.52 (3H, d, J = 4.0 Hz) |
| 14-OH |  | 7.90 (-OH,s) |
| 16-OH |  |  |

^a^ 125 MHz

^b^ 500 MHz

**Supplementary Table S6** | Results of drug-induced haploinsufficiency (DIH) screenings in *S. pombe* heterozygous deletion mutants of 94 genes related to posttranslational modification, protein turnover, and chaperones.

| *S. pombe*  Systemic ID | *S. pombe*  Gene name | *S. pombe* Gene Description | Rank | Fitness value (AVG) | STDEV |
| --- | --- | --- | --- | --- | --- |
| SP286 | - | Wild-type | - | 1.79 | 0.2487 |
| SPAC1002.14 | *itt1* | ubiquitin-protein ligase E3 involved in regulation of cytoplasmic translational termination (predicted) | 45 | 1.58 | 0.1820 |
| SPAC10F6.05c | *ubc6* | ubiquitin conjugating enzyme E2 Ubc6 (predicted) | 74 | 1.16 | 0.0543 |
| SPAC110.04c | *pss1* | heat shock protein Pss1 | 2 | 3.73 | 0.4700 |
| SPAC11E3.04c | *ubc13* | ubiquitin conjugating enzyme E2 Ubc13 | 50 | 1.41 | 0.1316 |
| SPAC11G7.02 | *pub1* | HECT-type ubiquitin-protein ligase E3 Pub1 | 87 | 1.04 | 0.0239 |
| SPAC1250.03 | *ubc14* | ubiquitin conjugating enzyme E2 for HECT-type and RBR family E3 Ub ligases, Ubc14 (predicted) | 66 | 1.20 | 0.2034 |
| SPAC12B10.01c | - | HECT-type ubiquitin-protein ligase E3, implicated in negative regulation of ubiquitinated chromatin formation (predicted) | 63 | 1.25 | 0.1413 |
| SPAC12G12.04 | *mcp60* | mitochondrial heat shock protein Hsp60/Mcp60 | 91 | 1.00 | 0.1175 |
| SPAC13A11.04c | *ubp8* | SAGA complex ubiquitin C-terminal hydrolase Ubp8 | 38 | 1.66 | 0.1195 |
| SPAC13G7.02c | *ssa1* | heat shock protein Ssa1 (predicted) | 23 | 1.93 | 0.3330 |
| SPAC1420.02c | *cct5* | chaperonin-containing T-complex epsilon subunit Cct5 | 15 | 2.08 | 0.4411 |
| SPAC167.07c | *hul5* | HECT-type ubiquitin-protein ligase E3 (predicted) | 40 | 1.64 | 0.2549 |
| SPAC17A2.04c | *cns1* | HSP chaperone complex subunit Cns1 (predicted) | 8 | 2.54 | 0.2465 |
| SPAC1805.15c | *pub2* | HECT-type ubiquitin-protein ligase E3 Pub2 | 84 | 1.07 | 0.1696 |
| SPAC1D4.04 | *cct2* | chaperonin-containing T-complex beta subunit Cct2 | 24 | 1.92 | 0.0752 |
| SPAC1F5.06 | *lsh1* | ER heat shock protein Lsh1 (predicted) | 53 | 1.37 | 0.1191 |
| SPAC20H4.10 | *ufd2* | ubiquitin-protein ligase E4 Ufd2 (predicted) | 12 | 2.23 | 0.3724 |
| SPAC22H10.10 | *alp21* | tubulin specific chaperone cofactor E | 51 | 1.38 | 0.1491 |
| SPAC23G3.08c | *ubp7* | ubiquitin C-terminal hydrolase Ubp7 | 7 | 2.57 | 0.4183 |
| SPAC24C9.14 | *otu1* | ubiquitin-specific cysteine protease, OTU family, Otu1 | 32 | 1.77 | 0.1852 |
| SPAC27F1.03c | *uch1* | ubiquitin C-terminal hydrolase Uch1 | 49 | 1.42 | 0.1979 |
| SPAC328.06 | *ubp2* | ubiquitin C-terminal hydrolase Ubp2 | 10 | 2.45 | 0.3458 |
| SPAC328.08c | *tbc1* | tubulin specific chaperone cofactor C, GTPase activating protein Tbc1 (predicted) | 73 | 1.17 | 0.1122 |
| SPAC343.18 | *rfp2* | SUMO-targeted ubiquitin-protein ligase subunit Rfp2 | 22 | 1.94 | 0.2025 |
| SPAC3A12.03c | *meu34* | ubiquitin-protein ligase E3 Meu34, human RNF13 family homolog, unknown biological role (predicted) | 26 | 1.90 | 0.0730 |
| SPAC3A12.12 | *atp11* | mitochondrial F1-FO ATP synthase chaperone Atp11 (predicted) | 25 | 1.91 | 0.0552 |
| SPAC57A7.12 | *ssz1* | heat shock protein Ssz1 (predicted) | 35 | 1.72 | 0.5155 |
| SPAC664.11 | *ssc1* | mitochondrial (2Fe-2S) cluster assembly chaperone Ssc1 | 62 | 1.27 | 0.1132 |
| SPAC6B12.07c | - | ubiquitin-protein ligase E3 with SPX domain, human LORNRF1 ortholog (predicted) | 93 | 0.96 | 0.0688 |
| SPAC6G9.08 | *ubp6* | ubiquitin C-terminal hydrolase Ubp6 | 33 | 1.76 | 0.0452 |
| SPAC8E11.07c | *alp31* | tubulin specific chaperone cofactor A, Alp31 | 72 | 1.17 | 0.0930 |
| SPAC926.04c | *hsp90* | Hsp90 chaperone | 1 | 4.16 | 0.7104 |
| SPAP32A8.03c | *bop1* | ubiquitin-protein ligase E3, human RNF126 ortholog (predicted) | 46 | 1.56 | 0.0256 |
| SPBC106.06 | *cct4* | chaperonin-containing T-complex delta subunit Cct4 | 9 | 2.51 | 0.2022 |
| SPBC1105.09 | *ubc15* | ubiquitin conjugating enzyme E2 Ubc15 | 55 | 1.33 | 0.0998 |
| SPBC119.02 | *ubc4* | ubiquitin conjugating enzyme E2 for APC and SCF Ubc4/UbcP1 | 19 | 1.99 | 0.1546 |
| SPBC1198.09 | *ubc16* | ubiquitin conjugating enzyme E2 Ubc16 (predicted) | 16 | 2.05 | 0.2918 |
| SPBC11C11.04c | *alp1* | tubulin specific chaperone cofactor D, Alp1 | 30 | 1.80 | 0.0680 |
| SPBC12D12.03 | *cct1* | chaperonin-containing T-complex alpha subunit Cct1 | 3 | 3.53 | 0.5575 |
| SPBC12D12.08c | *ned8* | ubiquitin-like protein modifier for cullin Ned8 | 52 | 1.37 | 0.0969 |
| SPBC14F5.10c | - | ubiquitin-protein ligase E3, unknown biological role, implicated in protein catabolic process (predicted) | 61 | 1.28 | 0.0404 |
| SPBC15C4.06c | - | ubiquitin-protein ligase E3 Meu34, human RNF13 family homolog, unknown biological role (predicted) | 60 | 1.28 | 0.1248 |
| SPBC16D10.08c | *hsp104* | heat shock protein Hsp104 | 18 | 2.03 | 0.1927 |
| SPBC16E9.11c | *pub3* | HECT-type ubiquitin-protein ligase E3 Pub3 (predicted) | 89 | 1.02 | 0.1717 |
| SPBC16G5.03 | *mrz1* | ubiquitin-protein ligase E3/SUMO transferase, Topors, possibly associated with DNA damage (predicted) | 79 | 1.14 | 0.0043 |
| SPBC1703.12 | *ubp9* | ubiquitin C-terminal hydrolase Ubp9 | 6 | 2.95 | 0.2333 |
| SPBC1709.05 | *sks2* | heat shock protein, ribosome associated molecular chaperone Sks2 | 56 | 1.32 | 0.0984 |
| SPBC17A3.10 | *pas4* | peroxisomal ubiquitin-protein ligase E3 involved in peroxisome organization and biogenesis (predicted) | 71 | 1.17 | 0.1004 |
| SPBC18H10.08c | *ubp4* | ubiquitin C-terminal hydrolase Ubp4 | 92 | 1.00 | 0.0281 |
| SPBC19C2.04c | *ubp11* | ubiquitin C-terminal hydrolase Ubp11 | 59 | 1.28 | 0.0473 |
| SPBC1A4.08c | *cct3* | chaperonin-containing T-complex gamma subunit Cct3 | 58 | 1.30 | 0.1784 |
| SPBC1E8.02 | - | ER ubiquitin family protein (predicted) | 28 | 1.87 | 0.3921 |
| SPBC211.07c | *ubc8* | ubiquitin conjugating enzyme E2 Ubc8 (predicted) | 57 | 1.32 | 0.1775 |
| SPBC21D10.09c | *rkr1* | RQC complex ubiquitin-protein ligase E3 Rkr1 (predicted) | 41 | 1.62 | 0.0729 |
| SPBC25H2.12c | *cct7* | chaperonin-containing T-complex eta subunit Cct7 | 5 | 3.08 | 0.1930 |
| SPBC26H8.14c | *cox17* | mitochondrial copper chaperone for cytochrome c oxidase Cox17 (predicted) | 64 | 1.22 | 0.0980 |
| SPBC28F2.08c | *hrd3* | Hrd1 ubiquitin ligase complex subunit (predicted) | 83 | 1.09 | 0.0120 |
| SPBC29A3.03c | *gid2* | GID complex ubiquitin-protein ligase E3 subunit Gid2/Rmd5 (predicted) | 34 | 1.72 | 0.2417 |
| SPBC2D10.20 | *ubc1* | ubiquitin conjugating enzyme Ubc1 (predicted) | 47 | 1.49 | 0.0455 |
| SPBC31E1.03 | *hub1* | ubiquitin-like protein modifier Hub1 | 70 | 1.18 | 0.1525 |
| SPBC337.05c | *cct8* | chaperonin-containing T-complex theta subunit Cct8 | 20 | 1.95 | 0.1186 |
| SPBC337.08c | *ubi4* | protein modifier, ubiquitin | 43 | 1.62 | 0.2407 |
| SPBC3B9.19 | *mge1* | mitochondrial [2Fe-2S] cluster assembly and protein import chaperone Mge1 (predicted) | 29 | 1.85 | 0.1284 |
| SPBC3D6.11c | *slx8* | SUMO-targeted ubiquitin-protein ligase E3 Slx8 | 37 | 1.66 | 0.0693 |
| SPBC3E7.02c | *hsp16* | heat shock protein Hsp16 | 67 | 1.20 | 0.0807 |
| SPBC409.05 | *skp1* | SCF ubiquitin ligase complex subunit Skp1 | 31 | 1.78 | 0.0949 |
| SPBC409.06 | *uch2* | ubiquitin C-terminal hydrolase Uch2 | 76 | 1.15 | 0.2179 |
| SPBC4F6.17c | *hsp78* | mitochondrial heatshock protein Hsp78 (predicted) | 13 | 2.09 | 0.2846 |
| SPBC646.11 | *cct6* | chaperonin-containing T-complex zeta subunit Cct6 | 4 | 3.21 | 0.5087 |
| SPBC6B1.06c | *ubp14* | Lys48-specific deubiquitinase Ubp14 | 75 | 1.15 | 0.1221 |
| SPBC713.02c | *ubp15* | ubiquitin C-terminal hydrolase Ubp15 | 27 | 1.90 | 0.1622 |
| SPBC947.10 | *dsc1* | Golgi Dsc E3 ligase complex subunit Dsc1 | 54 | 1.33 | 0.1975 |
| SPBP16F5.04 | *ubc7* | Hrd1 ubiquitin ligase complex ubiquitin conjugating enzyme E2 Ubc7 | 36 | 1.68 | 0.2581 |
| SPBP4H10.07 | - | ubiquitin-protein ligase E3, unknown specificity (predicted) | 86 | 1.04 | 0.1398 |
| SPBP8B7.15c | *mpe1* | mRNA cleavage ubiquitin-protein ligase E3 Mpe1 (predicted) | 81 | 1.09 | 0.0359 |
| SPBP8B7.21 | *ubp3* | ubiquitin C-terminal hydrolase Ubp3 | 69 | 1.19 | 0.1070 |
| SPBP8B7.23 | *rnf10* | ubiquitin-protein ligase E3 (predicted) | 21 | 1.95 | 0.1402 |
| SPBP8B7.27 | *mug30* | HECT-type ubiquitin-protein ligase E3, found in association with TRAMP complex (predicted) | 68 | 1.19 | 0.2106 |
| SPCC1223.01 | - | ubiquitin-protein ligase E3 involved in rescue of stalled ribosomes (predicted) | 39 | 1.65 | 0.3306 |
| SPCC1259.15c | *ubc11* | ubiquitin conjugating enzyme E2, Ubc11/UbcP4 | 42 | 1.62 | 0.1249 |
| SPCC1494.05c | *ubp12* | CSN-associated deubiquitinating enzyme Ubp12 | 65 | 1.21 | 0.1426 |
| SPCC1682.12c | *ubp16* | ubiquitin C-terminal hydrolase Ubp16 | 90 | 1.02 | 0.1373 |
| SPCC16A11.12c | *ubp1* | ubiquitin C-terminal hydrolase Ubp1 | 44 | 1.60 | 0.1343 |
| SPCC1739.13 | *ssa2* | heat shock protein Ssa2 | 78 | 1.15 | 0.1174 |
| SPCC188.08c | *ubp5* | ubiquitin C-terminal hydrolase Ubp5 | 77 | 1.15 | 0.0215 |
| SPCC338.05c | *mms2* | ubiquitin conjugating enzyme Mms2 | 48 | 1.43 | 0.1034 |
| SPCC338.06c | - | heat shock protein Hsp20 family (predicted) | 11 | 2.37 | 0.3001 |
| SPCC4G3.12c | - | ubiquitin-protein ligase E3 (predicted) | 88 | 1.03 | 0.0371 |
| SPCC548.04 | *urm1* | ubiquitin-like protein modifier Urm1 (predicted) | 85 | 1.04 | 0.1067 |
| SPCC548.05c | *dbl5* | ubiquitin-protein ligase E3 Dbl5 | 14 | 2.09 | 0.1323 |
| SPCC550.06c | *hsp10* | mitochondrial heat shock protein Hsp10 (predicted) | 94 | 0.96 | 0.0938 |
| SPCC645.14c | *sti1* | chaperone activator Sti1 (predicted) | 80 | 1.14 | 0.2000 |
| SPCC777.10c | *ubc12* | NEDD8-conjugating enzyme Ubc12 | 82 | 1.09 | 0.0490 |
| SPCC970.10c | *brl2* | ubiquitin-protein ligase E3 Brl2 | 17 | 2.04 | 0.0689 |
